# PD-L1 translocation to the plasma membrane enables tumor immune evasion through MIB2 ubiquitination

**DOI:** 10.1172/JCI160456

**Published:** 2023-02-01

**Authors:** Xinfang Yu, Wei Li, Haidan Liu, Xu Wang, Cristian Coarfa, Chao Cheng, Xinlian Yu, Zhaoyang Zeng, Ya Cao, Ken H. Young, Yong Li

**Affiliations:** 1 Department of Medicine, Dan L Duncan Comprehensive Cancer Center, Baylor College of Medicine, Houston, Texas, USA.; 2 Department of Radiology, The Third Xiangya Hospital of Central South University, Changsha, Hunan, China.; 3 Department of Cardiovascular Surgery, The Second Xiangya Hospital of Central South University, Changsha, Hunan, China.; 4 Clinical Center for Gene Diagnosis and Therapy, The Second Xiangya Hospital of Central South University, Changsha, Hunan, China.; 5 Department of Molecular Cell Biology, Dan L Duncan Comprehensive Cancer Center, Baylor College of Medicine, Houston, Texas, USA.; 6 School of Transportation, Southeast University, Nanjing, Jiangsu, China.; 7 Key Laboratory of Carcinogenesis and Invasion, Chinese Ministry of Education, Xiangya Hospital, Central South University, Changsha, Hunan, China.; 8 Department of Pathology, Division of Hematopathology, Duke University Medical Center, Durham, North Carolina, USA.

**Keywords:** Cell Biology, Oncology, Cancer, Cancer immunotherapy, Cellular immune response

## Abstract

Programmed death-ligand 1 (PD-L1), a critical immune checkpoint ligand, is a transmembrane protein synthesized in the endoplasmic reticulum of tumor cells and transported to the plasma membrane to interact with programmed death 1 (PD-1) expressed on T cell surface. This interaction delivers coinhibitory signals to T cells, thereby suppressing their function and allowing evasion of antitumor immunity. Most companion or complementary diagnostic devices for assessing PD-L1 expression levels in tumor cells used in the clinic or in clinical trials require membranous staining. However, the mechanism driving PD-L1 translocation to the plasma membrane after de novo synthesis is poorly understood. Herein, we showed that mind bomb homolog 2 (MIB2) is required for PD-L1 transportation from the *trans*-Golgi network (TGN) to the plasma membrane of cancer cells. MIB2 deficiency led to fewer PD-L1 proteins on the tumor cell surface and promoted antitumor immunity in mice. Mechanistically, MIB2 catalyzed nonproteolytic K63-linked ubiquitination of PD-L1, facilitating PD-L1 trafficking through Ras-associated binding 8–mediated (RAB8-mediated) exocytosis from the TGN to the plasma membrane, where it bound PD-1 extrinsically to prevent tumor cell killing by T cells. Our findings demonstrate that nonproteolytic ubiquitination of PD-L1 by MIB2 is required for its transportation to the plasma membrane and tumor cell immune evasion.

## Graphical abstract

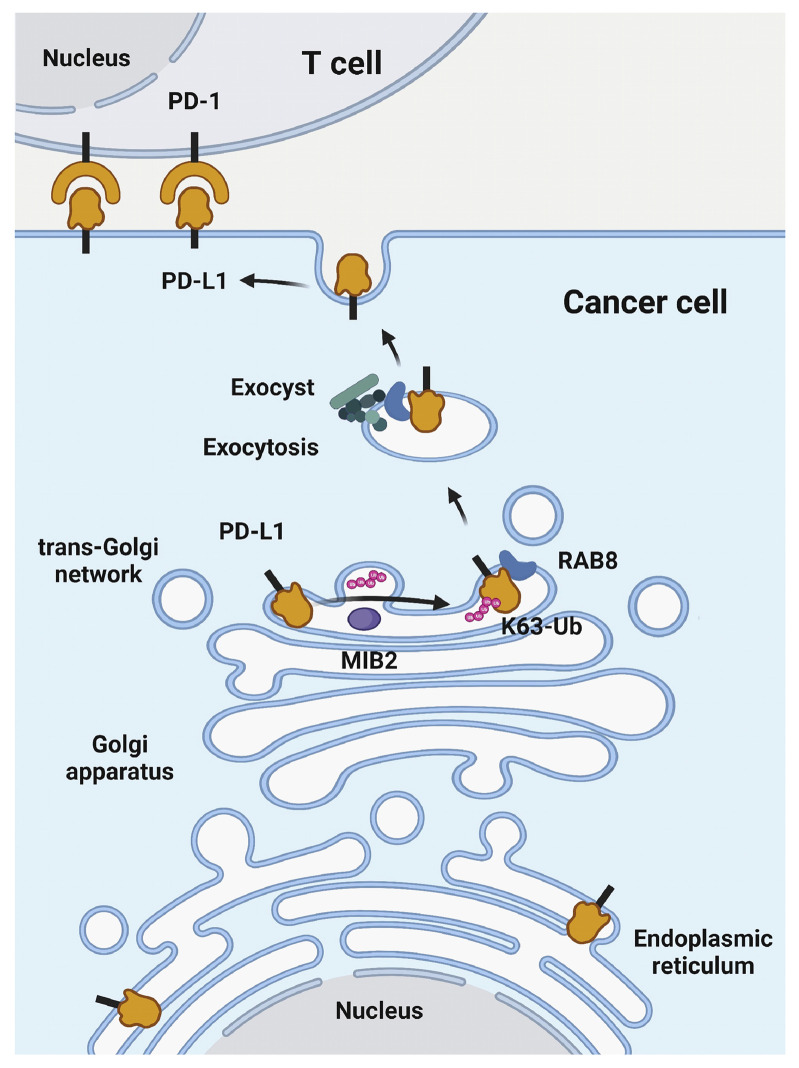

## Introduction

Programmed death-ligand 1 (PD-L1, also known as CD274 or B7-H1) is a ligand of immunosuppressive checkpoint programmed death 1 (PD-1) and is overexpressed in various cancers (1, 2). Although PD-L1 has a broad distribution in different cellular compartments, including the plasma membrane (3, 4), cytoplasm (3), and nucleus (5), or is secreted into the circulation (6), membrane-bound PD-L1 appears to be particularly important for its extrinsic function. PD-L1 expressed on the tumor cell plasma membrane binds its receptor, PD-1, on the T cell surface to inhibit the tumor-killing activity of cytotoxic T lymphocytes (CTLs). Immune checkpoint blockade (ICB) with antibodies to inhibit the interaction between PD-1 and PD-L1 has opened a new era for cancer therapy (7). However, considerable challenges, such as the lack of durable remission, low response rate, and drug resistance, limit therapeutic benefits to a small fraction of patients with cancer. Induction of PD-L1 expression in tumor cells is central to adaptive immune resistance (8). The regulation of membrane PD-L1 stability is implicated in tumor immune surveillance; in tumors, its increased degradation augments tumor-specific T cell activity (9). In addition, intracellular PD-L1 is translocated onto the plasma membrane to restore the membrane PD-L1 pool, facilitating tumor immune evasion in the tumor microenvironment (10). Therefore, increasing our understanding of the regulation of PD-L1 translocation to the plasma membrane will provide novel therapeutic opportunities to enhance ICB.

Transmembrane proteins frequently shuttle between the plasma membrane and the endomembrane system, which consists of the endoplasmic reticulum (ER), Golgi apparatus, and lysosomes. PD-L1 spatiotemporal distribution and dynamics at the plasma membrane are tightly regulated (11). Nascent PD-L1 proteins synthesized in the rough ER are subjected to posttranslational modifications (PTMs). STT3A and STT3B mediate N-glycosylation of PD-L1 on the ER, stabilizing and upregulating PD-L1 inside the cell (12), whereas B3GNT3 mediates PD-L1 glycosylation to help its interaction with PD-1 on the cell surface (13). The thyroid adenoma-associated gene (THADA) regulates PD-L1–specific ER export to the Golgi by coupling PD-L1 to Sec24A-mediated COP2 vesicles, thereby maintaining the PD-L1 Golgi residency and tumor cell expression (14). Ubiquitination plays a dominant role in regulating proteasome-mediated degradation of PD-L1. STUB1 (15), β-TRCP (16), SPOP (17), ARIH1 (18), TRAF6 (19), TRIM21 (20), MARCH8 (21), SIAH2 (22), FBXO22 (23), TNFAIP3 (also known as A20) (24), and ITCH (25) add ubiquitin chains to PD-L1, which are removed by CSN5 (26), USP9X (27), USP22 (28), USP8 (19), and OTUB1 (29, 30). PD-L1 phosphorylation mediated by GSK3A (18), GSK3Β (16), or AMPK (31, 32) precedes its glycosylation and may lead its ER accumulation and ER-associated protein degradation. Recycling of PD-L1 is regulated by its PTMs. DHHC3/ZDHHC9-mediated palmitoylation blocks PD-L1 ubiquitination and endosomal sorting complexes required for transport-mediated sorting to multivesicular body/lysosomes (33, 34). The chemokine-like factor–like MARVEL transmembrane domain-containing family members 6 and 4 (CMTM6 and CMTM4) colocalize with and maintain PD-L1 at the plasma membrane and in recycling endosomes, rescuing PD-L1 from lysosomal degradation (15, 32, 35). TRAPPC4 maintains PD-L1 levels by regulating the endosomal recycling of PD-L1, protecting PD-L1 from lysosomal degradation, and ultimately impairing T cell–mediated antitumor immunity (36). PD-L1 is one of the few plasma membrane proteins that enters the nucleus, a process that is regulated by acetylation, another form of PTM. PD-L1 translocation from the plasma membrane into the nucleus is blocked by p300-mediated acetylation, which is enhanced upon its deacetylation by HDAC2 (5). These findings suggest that PTMs regulate both the extrinsic and intrinsic functions of PD-L1 by controlling its trafficking to different cellular compartments. However, PTMs that direct PD-L1 trafficking to the plasma membrane have not been reported.

In this study, we found that mind bomb homolog 2–mediated (MIB2-mediated) K63-linked ubiquitination of PD-L1 facilitates its translocation from the *trans*-Golgi network (TGN) to the plasma membrane through RAB8-regulated exocytosis, ultimately promoting immune evasion and tumor progression. This study provides what we believe to be new insights into the molecular regulation of PD-L1 transportation to the surfaces of cancer cells, which may help identify new avenues to improve clinical outcomes from ICB therapies.

## Results

### MIB2 maintains PD-L1 membrane abundance and compromises antitumor immunity.

**Figure 1:**
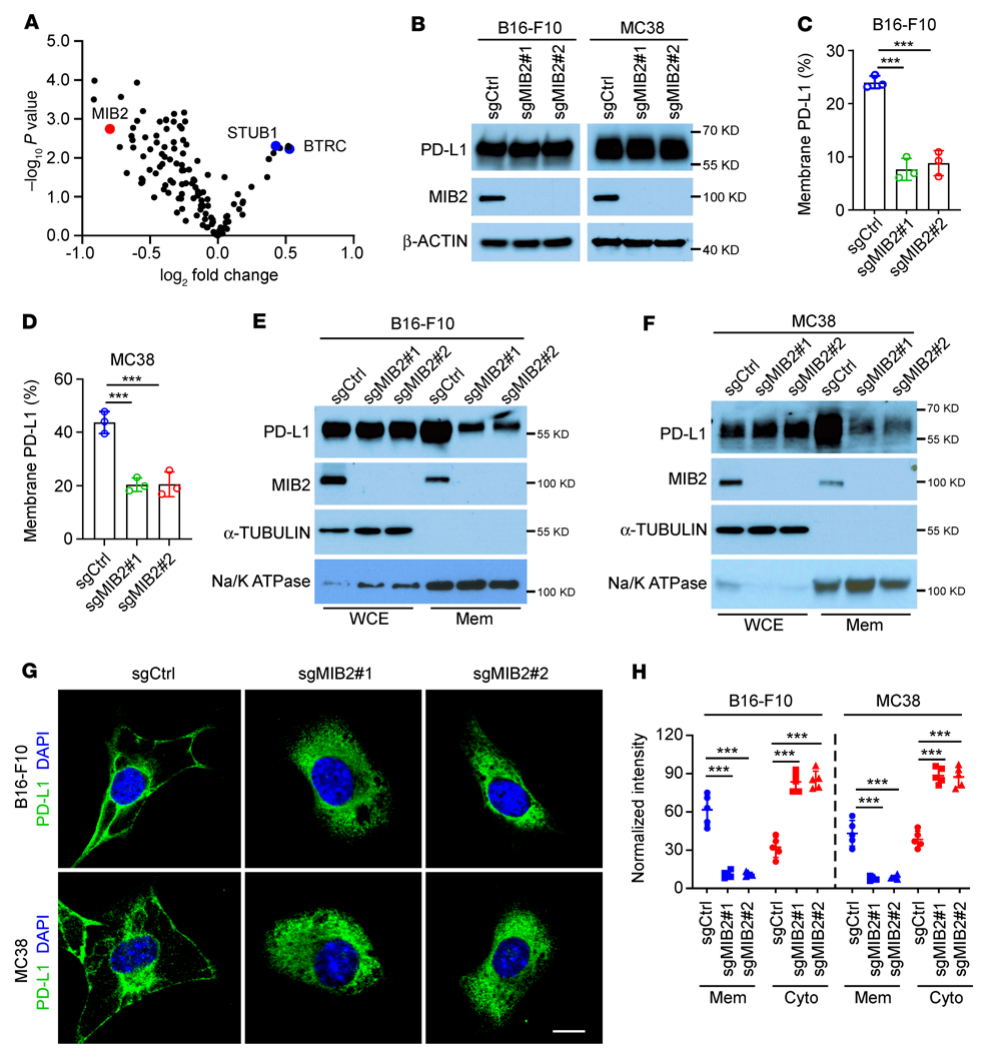
MIB2 regulates membrane PD-L1 levels in tumor cells. (**A**) Volcano plot showing the E3 ligases identified from FACS. MIB2 is indicated in red, and STUB1 and BTRC are indicated in blue. (**B**) Immunoblotting (IB) analysis of PD-L1 levels in MIB2-KO B16-F10 and MC38 cells. (**C** and **D**) FACS analysis of membrane PD-L1 levels in (**C**) B16-F10 and (**D**) MC38 cells. (**E** and **F**) IB analysis of PD-L1 protein levels in whole-cell extract (WCE) and membrane fractions (Mem) from (**E**) B16-F10 and (**F**) MC38 cells. (**G** and **H**) Immunofluorescence analysis of PD-L1 in B16-F10 and MC38 cells. (**G**) Representative images of green fluorescence–labeled PD-L1. Scale bar: 10 μm. (**H**) Quantitative analysis of membrane- and cytoplasmic-expressed PD-L1 (*n* = 5). Cyto, cytoplasm. ***P < 0.001 by 1-way ANOVA test with Dunnett’s multiple comparisons test (**C**, **D**, and **H**).

We performed an E3 ligase knockdown screening in human A375 cells with an shRNA library consisting of pooled oligos (a mixture of 4 shRNAs per E3 ligase) targeting 137 human E3 ligases (Supplemental Table 1; supplemental material available online with this article; https://doi.org/10.1172/JCI160456DS1). Membrane PD-L1 levels were examined using FACS analysis. Knockdown of E3 ligases for PD-L1 degradation, including *STUB1* (15) and *TRCP* (*BTRC*) (16), significantly upregulated membrane PD-L1 (Figure 1A and Supplemental Figure 1A). *MIB2*, *TRIM3*, *BARD1*, *FBW7*, and *RBX1* were the top 5 candidate E3 ligases with knockdown that led to reduced PD-L1 membrane abundance (Figure 1A and Supplemental Figure 1, A and B). Unlike that of the other 4 E3 ligases, *MIB2* knockdown in human A375 and A549 cells did not reduce the total protein level of PD-L1 in the whole-cell extract (Supplemental Figure 1C). FACS and immunoblotting (IB) data showed that KO of *MIB2* by CRISPR in mouse B16-F10, MC38, and LLC1 cells (Figure 1, B–D, and Supplemental Figure 1D) decreased PD-L1 membrane abundance but exhibited no apparent effect on PD-L1 total protein levels. We further confirmed our observations in the other 4 human cell lines, A549, HT29, HCT116, and A375 (Supplemental Figure 1, E–H). Cellular fractionation assays revealed that MIB2 depletion decreased membrane PD-L1 levels in B16-F10, MC38, LLC1, and A375 cells (Figure 1, E and F, and Supplemental Figure 1, I and J). Immunofluorescence (IF) analysis revealed that PD-L1 was located primarily on the plasma membranes and around the nuclei in MIB2-proficient B16-F10 and MC38 cells (Figure 1, G and H). However, in *MIB2*-KO cells, PD-L1 was detected with an increased signal around the nuclei, but it was no longer detected on the plasma membrane (Figure 1, G and H). As membrane proteins are shuttled from the ER to Golgi before they reach the plasma membrane, we costained PD-L1 with markers for ER (HSP90B1) or Golgi (TGN46) in MIB2-proficient and MIB2-KO A375 cells (Supplemental Figure 1K). PD-L1 colocalized with HSP90B1 and TGN46, but its plasma membrane localization disappeared upon *MIB2* KO. These results suggest that MIB2 is required for PD-L1 trafficking to the plasma membrane in human and mouse cancer cells.

**Figure 2:**
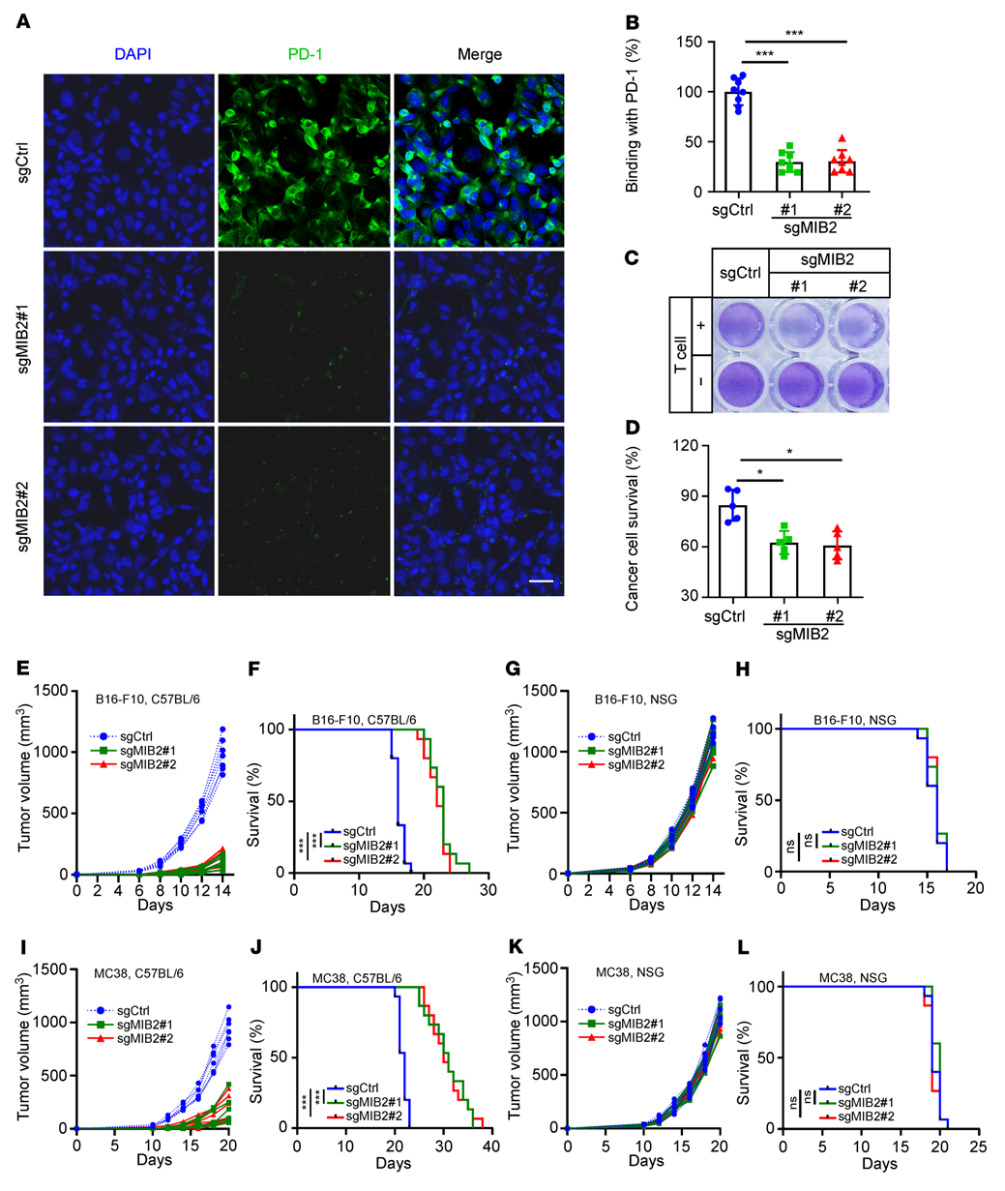
Depletion of MIB2 enhances antitumor immunity. (**A** and **B**) Immunofluorescence analysis of PD-1 at the A375 cell surface. (**A**) Representative images of binding of green fluorescence–labeled exogenous PD-1-Fc. Scale bar: 25 μm. (**B**) Quantitation (*n* = 7). (**C** and **D**) A375 cell survival upon incubation with allogeneic T cells. A375 cells were cocultured with or without activated T cells at a ratio of 1:5 for 48 hours and subjected to crystal violet staining. (**C**) Representative images. (**D**) Quantitation. (**E**) Growth curve of B16-F10 tumors in C57BL/6 mice (*n* = 7 per group). (**F**) Kaplan-Meier survival curves of B16-F10 tumor-bearing C57BL/6 mice (*n* = 15 per group). (**G**) Growth curve of B16-F10 tumors in NSG mice (*n* = 7 per group). (**H**) Kaplan-Meier survival curves of B16-F10 tumor-bearing NSG mice (*n* = 15 per group). (**I**) Growth curve of MC38 tumors in C57BL/6 mice (*n* = 7 per group). (**J**) Kaplan-Meier survival curves of MC38 tumor-bearing C57BL/6 mice (*n* = 15 per group). (**K**) Growth curve of MC38 tumors in NSG mice (*n* = 7 per group). (**L**) Kaplan-Meier survival curves of MC38 tumor-bearing NSG mice (*n* = 15 per group). *P < 0.05; ***P < 0.001; by log-rank (Mantel-Cox) test (**F**, **H**, **J**, and **L**) and by 1-way ANOVA test with Dunnett’s multiple comparisons test (**B** and **D**). Data are shown as the mean ± SEM.

When incubated with recombinant human PD-1-Fc fusion protein, we found that *MIB2* KO led to decreased PD-1 binding in A375 cells (Figure 2, A and B). Furthermore, *MIB2* KO enhanced T cell–mediated tumor cell killing in vitro (Figure 2, C and D). Reintroduction of MIB2 in *MIB2*-KO A375 cells restored PD-1 binding (Supplemental Figure 2A) and impaired T cell–mediated tumor cell killing in vitro (Supplemental Figure 2B). We found that the depletion of MIB2 in B16-F10 cells caused significant tumor regression and prolonged survival in immunocompetent C57BL/6 syngeneic mice but not in immunodeficient NOD/SCID IL2rg^null^ (NSG) mice (Figure 2, E–H, and Supplemental Figure 2C). Consistently, the loss of *MIB2* in MC38 (Figure 2, I–L, and Supplemental Figure 2D) and LLC1 (Supplemental Figure 2, E–I) cells only restricted tumor development and improved survival in C57BL/6 mice. The in vivo metastasis models showed that B16-F10 single-guide control (sgCtrl) and single-guide MIB2 (sgMIB2) cells exhibited similar metastatic efficacy in NSG mice (Supplemental Figure 2, J–N). However, the metastatic process was delayed in C57BL/6 mice, as *MIB2*-KO B16-F10 cells showed a significant decrease in lung weight and the number/diameter of metastatic lung nodules and prolonged overall survival (Supplemental Figure 2, O–S). In particular, *MIB2* KO in combination with the reintroduction of *MIB2* rescued the growth of B16-F10 tumors and reduced survival time in C57BL/6 mice (Supplemental Figure 3, A–C). However, tumor development and mouse survival rate appeared to be comparable in NSG mice regardless of MIB2 status (Supplemental Figure 3, D–F). Similar results from MIB2-restored MC38 tumors were observed in C57BL/6 and NSG mice (Supplemental Figure 3, G–L). These data indicate that loss of MIB2 reduces membrane PD-L1 levels and activates extrinsic antitumor immunity in immunocompetent mice, without having an intrinsic effect on tumorigenesis in an immunodeficient environment.

To better understand the mechanisms by which MIB2 downregulation via shRNAs reactivates antitumor immunity, we performed single-cell RNA-Seq (scRNA-Seq) of B16-F10 tumors from C57BL/6 syngeneic mice. Seven types of cells were identified in the whole-tumor microenvironment, including B16-F10 cells, T cells, NK cells, stromal cells, mononuclear phagocyte system cells, B cells, and erythrocytes (Supplemental Figure 4, A and B). Notably, the populations of the total immune cells increased in MIB2-shRNA tumors compared with those in the shCtrl group (Supplemental Figure 4C). We determined whether *MIB2* knockdown affected T cell–mediated antitumor immune activity by characterizing the subpopulations of tumor-infiltrating T cells. A total of 6 distinct T cell subpopulations, including NKT cells, CD4^+^CD8^+^ effector T cells, CD8^+^ effector/activated T cells, CD4^+^CD8^+^ naive T cells, CD8^+^ CTLs, and CD4^+^ regulatory T cells, were identified based on the distribution of classical markers (Supplemental Figure 4, D–G). Knockdown of *MIB2* resulted in an increase in the percentage of CD8^+^ CTLs (approximately 5-fold) and CD4^+^CD8^+^ effector/activated T cells (approximately 2-fold) (Supplemental Figure 4F), indicating that MIB2 downregulation enhanced the antitumor immune activity centered on CD8^+^ CTLs and changed their transcriptional profile (Supplemental Figure 4H).

In line with the scRNA-Seq data, IF staining results revealed that *MIB2* knockdown increased the tumor-infiltrated CD8^+^ T cell population and granzyme B release (Supplemental Figure 4I) in B16-F10 tumors from C57BL/6 syngeneic mice. To further examine whether CD8^+^ T cells are required for antitumor immunity in MIB2-deficient tumors, we treated mice with neutralizing mAbs against CD8. CD8^+^ T cell depletion restored the growth of *MIB2*-KO B16-F10 tumors and significantly increased tumor burden (Supplemental Figure 4, J and K), reinforcing that the antitumor effect partly depends on T cells. Overall, these results suggest that MIB2 deficiency activates antitumor immunity and that CD8^+^ T cells contribute to an enhanced antitumor response in vivo.

### Depletion of MIB2 enhances the efficacy of immunotherapy.

**Figure 3:**
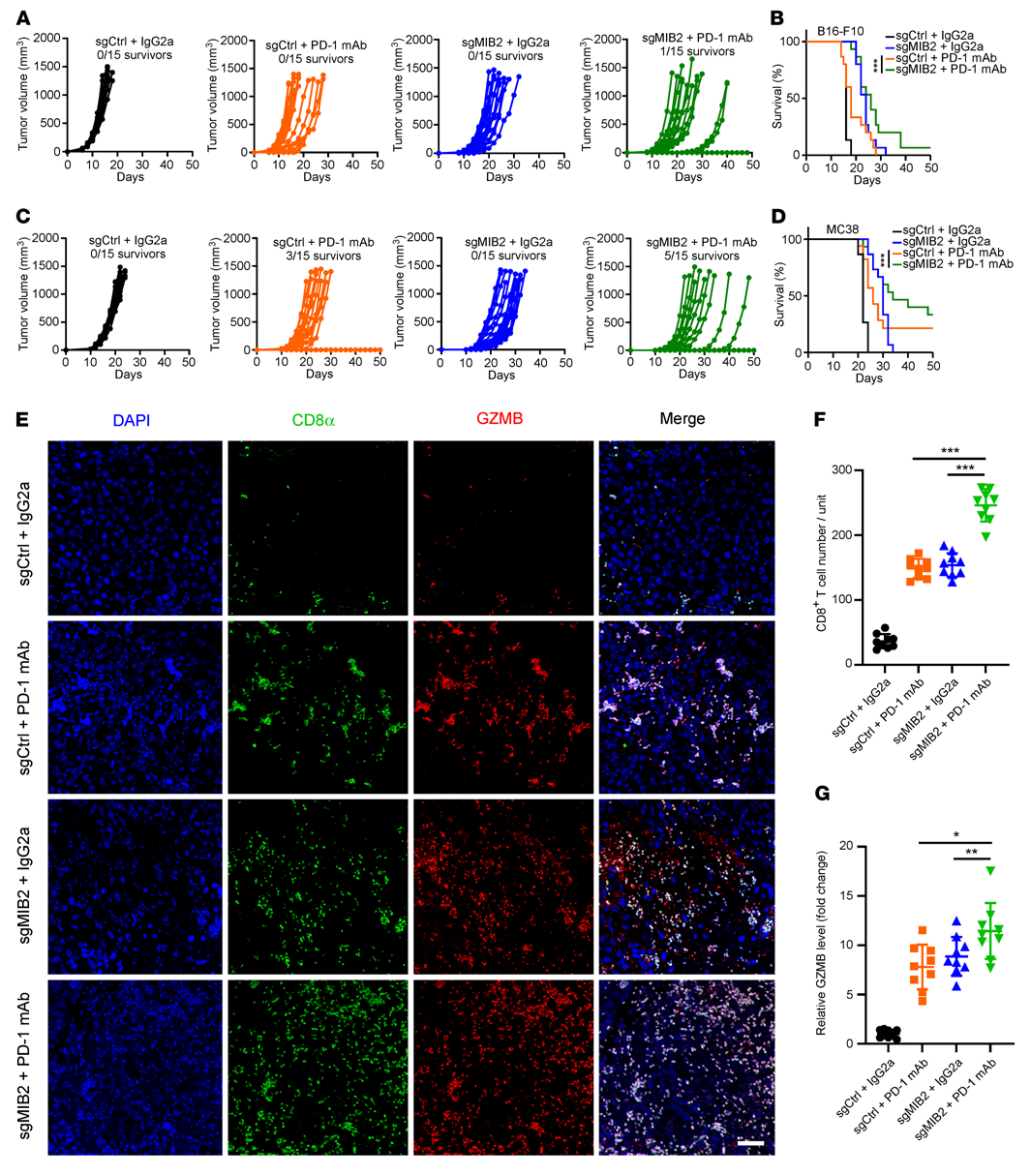
Depletion of MIB2 improves the efficacy of anti–PD-1 immunotherapy. (**A**) Volumes of B16-F10 syngeneic tumors treated with control antibody (IgG2a) or PD-1 mAb. (**B**) Kaplan-Meier survival curves for each treated group from **A** (*n* = 15 per group). (**C**) Volumes of MC38 syngeneic tumors treated with control antibody (IgG2a) or PD-1 mAb. (**D**) Kaplan-Meier survival curves for each treated group from **C** (*n* = 15 per group). (**E**–**G**) Immunostaining of CD8 and granzyme B (GZMB) in the B16-F10 tumors treated with control (IgG2a) or PD-1 mAb. Data are shown as the mean ± SD (*n* = 9); 3 tissue slides per tumor. (**E**) Representative images. Scale bar: 50 μm. (**F**) Quantification of CD8^+^ T cells. (**G**) Relative GZMB level. Unit = 262,144 μm^2^ (the area of the tumor tissue). *P < 0.05; **P < 0.01; ***P < 0.001 by log-rank (Mantel-Cox) test (**B** and **D**) and by 1-way ANOVA test with Dunnett’s multiple comparisons test (**F** and **G**). Data are shown as the mean ± SEM.

Based on our findings that MIB2 promotes membrane PD-L1 levels and compromises immune surveillance in vivo, we speculated that *MIB2* depletion might be a potential strategy for enhancing the efficacy of immunotherapy. We first treated B16-F10 and MC38 syngeneic mice with PD-1 mAb. *MIB2* KO delayed tumor development in both B16-F10 tumors with IgG isotype control antibody (IgG2A) treatment (Figure 3A). Although PD-1 mAb treatment slowed the growth of B16-F10 tumors, no mice survived for more than 30 days after tumor inoculation (Figure 3A). Notably, *MIB2* KO significantly enhanced the antitumor efficacy of PD-1 mAb, in that tumor growth was restricted and survival was improved (Figure 3, A and B). Similar antitumor efficacy was also observed in MC38 syngeneic mice. Treatment with PD-1 mAb markedly inhibited the growth of MIB2 WT MC38 tumors, and 3 of 15 of the tumor-bearing mice survived for more than 50 days after tumor inoculation (Figure 3C). The antitumor potential of PD-1 mAb was further enhanced in *MIB2*-KO MC38 tumors, in that the in vivo tumor development was substantially impaired, and 5 of 15 tumor-bearing mice were tumor-free for 50 days (Figure 3D). We found that *MIB2* KO further increased the population of CD8^+^ T cells and the expression level of granzyme B in PD-1 mAb–treated tumors (Figure 3, E–G). We next determined whether *MIB2* KO affects anti–PD-L1 or anti–CTLA-4 immunotherapies. Treatment with PD-L1 or CTLA4 mAb alone exhibited no significant delay in the growth of B16-F10 tumors (Supplemental Figure 5, A and C). *MIB2* KO markedly restricted tumor growth and improved survival rate when combined with either PD-L1 or CTLA4 mAb (Supplemental Figure 5, B and D), but no mice survived more than 50 days after tumor inoculation. These results indicate that depletion of MIB2 enhances the therapeutic benefit of immune checkpoint inhibitors.

### MIB2 promotes PD-L1 K63-linked polyubiquitination.

**Figure 4:**
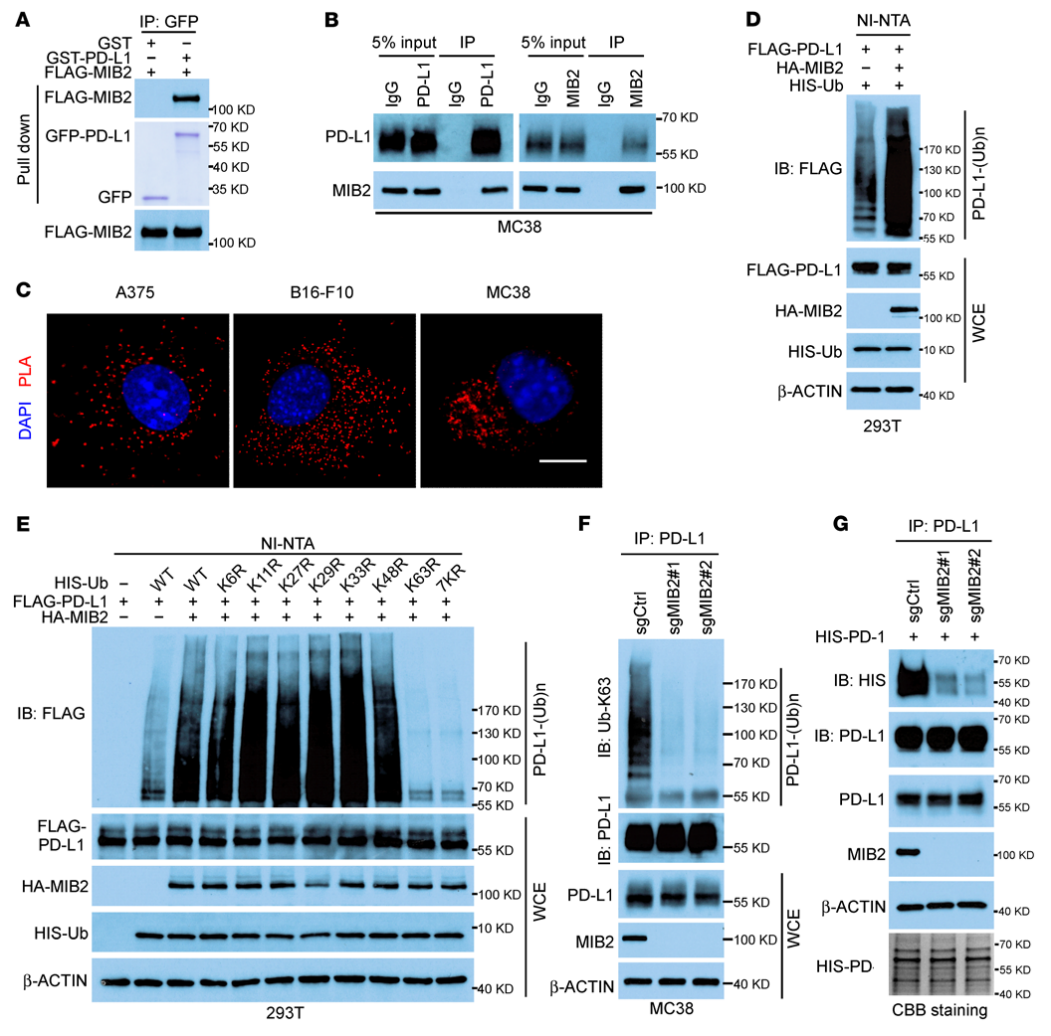
MIB2 promotes K63-linked ubiquitination of PD-L1. (**A**) Direct binding between PD-L1 and MIB2. FLAG-MIB2 immunoprecipitated from 293T cells bound to purified GST–PD-L1 in vitro. Purified GST–PD-L1 was examined by Coomassie Blue staining. (**B**) Co-IP analysis of the endogenous PD-L1 and MIB2 in MC38 cells. (**C**) Proximity ligation assay (PLA) analysis of PD-L1 and MIB2 in A375, B16-F10, and MC38 cells. Scale bar: 10 μm. (**D**) In vivo PD-L1 ubiquitination by MIB2 in 293T cells transfected with expression constructs. (**E**) Immunoprecipitation (IP) and immunoblotting (IB) analysis of 293T cells transfected with various ubiquitin mutant constructs. (**F**) IP and IB analysis of MIB2-KO MC38 cells using Ub-K63–specific antibody. (**G**) IP and IB analysis of PD-L1 in A375 cells interacting with purified HIS–PD-1.

To determine the underlying mechanism by which MIB2 maintains membrane PD-L1 levels, we first investigated whether MIB2 is a PD-L1–binding protein. Coimmunoprecipitation (co-IP) data revealed the interaction between MIB2 and PD-L1 in 293T cells ectopically overexpressing MIB2 and PD-L1 (Supplemental Figure 6A). In a previous study, mass spectrometry data also validated the interaction of PD-L1 with MIB2 (5). The interaction was confirmed in vitro using purified proteins (Figure 4A). The endogenous co-IP assay (Figure 4B and Supplemental Figure 6, B and C) and in situ proximity ligation assays (PLAs) (Figure 4C) revealed an endogenous binding of MIB2 and PD-L1 in A375, B16-F10, and MC38 cells. Deleting the extracellular domain (ECD) of PD-L1 reduced PD-L1–MIB2 interaction in 293T cells (Supplemental Figure 6, D and E). Furthermore, only the ECD mutant, but not the signal peptide domain, transmembrane domain, or intracellular domain mutant of PD-L1, exhibited binding with WT MIB2 (Supplemental Figure 6, F and G), indicating that the ECD is required for the PD-L1 and MIB2 interaction. Similarly, the co-IP assay using WT PD-L1 and truncated mutants of MIB2 suggested that the ankyrin repeat domain of MIB2 is essential for its binding with PD-L1 (Supplemental Figure 6, H and I).

Next, we determined whether PD-L1 is a substrate of E3 ligase MIB2. Cotransfection with MIB2 promoted PD-L1 polyubiquitination in 293T cells (Figure 4D). However, ectopic overexpression of MIB2 did not affect PD-L1 levels in A375 and MC38 cells (Supplemental Figure 7A). PD-L1 in MC38 cells with sgCtrl or sgMIB2 exhibited a similar half-life (Supplemental Figure 7B), indicating that MIB2 did not affect PD-L1 degradation. The K63R (K63 mutated to R) and 7KR (all K residues mutated to R) ubiquitin mutants reduced MIB2-mediated PD-L1 ubiquitination in 293T cells (Figure 4E). Consistently, ubiquitination analysis using the K63-linkage specific polyubiquitin antibody confirmed that PD-L1 ubiquitination by MIB2 is K63 linked (Supplemental Figure 7C). Deletion of the C-terminal RING domain required for MIB2 E3 ligase activity abolished PD-L1 ubiquitination (Supplemental Figure 7D), suggesting that MIB2-induced PD-L1 K63-linked ubiquitination is dependent on MIB2 E3 ligase activity. The in vitro ubiquitination assay showed that purified WT MIB2, but not the RING domain deletion mutant, ubiquitinated recombinant PD-L1, supporting that MIB2 is a bona fide E3 ligase for PD-L1 (Supplemental Figure 7E). MIB2 KO impaired endogenous K63-linked ubiquitination of PD-L1 in MC38, B16-F10, LLC1, and A375 cells (Figure 4F and Supplemental Figure 7F). In B16-F10 tumors from C57BL/6 mice, *MIB2* KO markedly reduced the endogenous K63-linked ubiquitination of PD-L1 (Supplemental Figure 7G). Moreover, the interaction between purified PD-1 protein and PD-L1 in *MIB2*-KO A375 cells was markedly reduced (Figure 4G), indicating that MIB2-induced ubiquitination is required for the interaction of PD-L1 and PD-1.

The activity of E3 ligases is often regulated by PTMs, including phosphorylation, sumoylation, ubiquitination, and binding of alternative adaptor molecules and cofactors (37–39). CDK1 is predicted to phosphorylate MIB2 by Scansite 4.0 (40). A CDK1 inhibitor (riviciclib) reduced MIB2 phosphorylation on Ser/Thr in B16-F10 cells (Supplemental Figure 7H). Autoubiquitination is a marker for the activation of some E3 ligases. Riviciclib inhibited the K63-linked autoubiquitination of MIB2 in B16-F10 cells (Supplemental Figure 7H). Moreover, PD-L1 membrane abundance and the K63-linked ubiquitination of PD-L1 were reduced by riviciclib treatment (Supplemental Figure 7, I and J). Thr105 (T105), a highly conserved residue on MIB2 (Supplemental Figure 7K), is a predicted phosphorylation site by CDK1. We constructed the MIB2 T105A mutant and performed IB and ubiquitination assays in 293T cells. The Ser/Thr phosphorylation was decreased in the T105A mutant (Supplemental Figure 7L), along with the K63-linked ubiquitination of PD-L1 by MIB2 and the PD-L1 membrane protein level (Supplemental Figure 7, L and M). These results indicate that E3 ligase activity of MIB2 in governing PD-L1 K63 ubiquitination and membrane localization is activated by CDK1.

### Ubiquitination of PD-L1 K136 induces its plasma membrane localization and impairs antitumor immunity.

**Figure 5:**
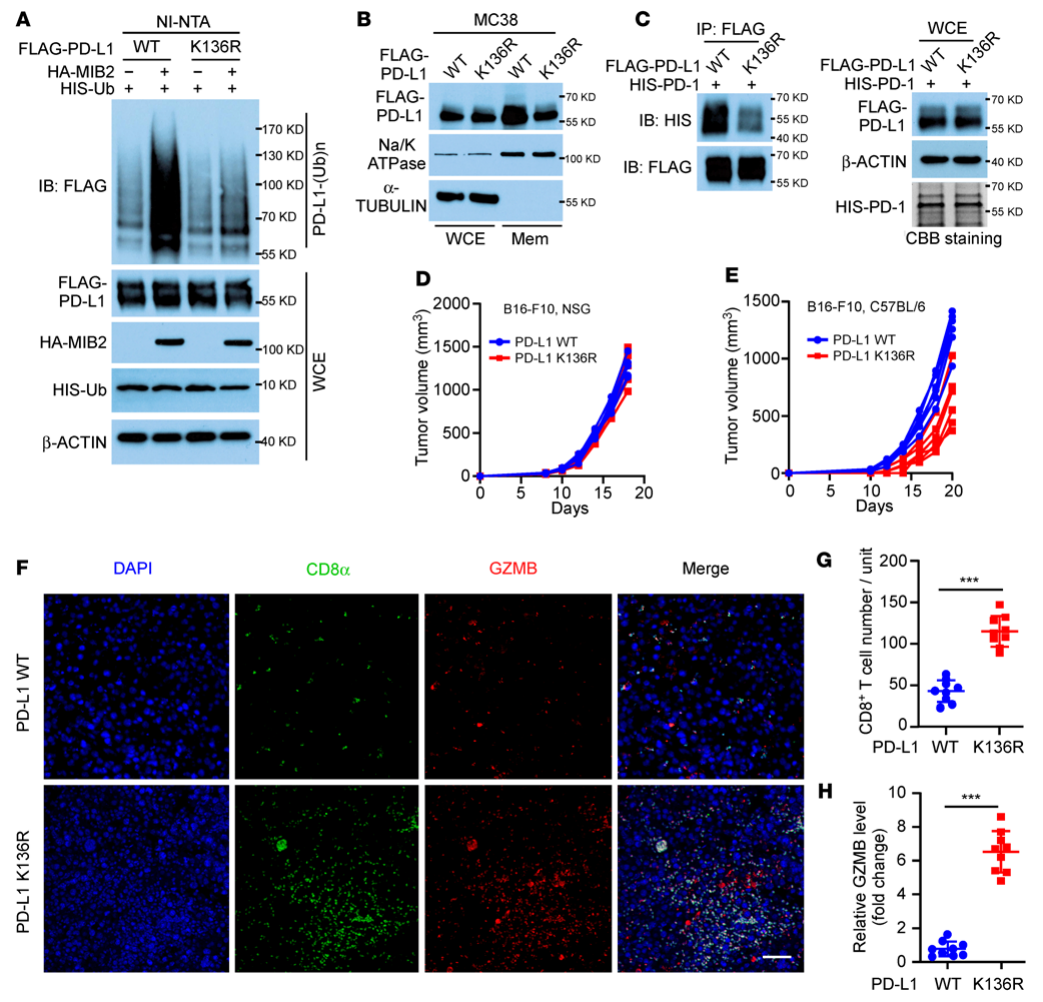
MIB2 catalyzes PD-L1 ubiquitination on K136 residue. (**A**) In vivo PD-L1 ubiquitination by MIB2 in 293T cells transfected with PD-L1 WT or the K136R mutant expression construct. (**B**) Immunoblotting (IB) analysis of PD-L1 protein levels in whole-cell extract (WCE) and membrane fractions (Mem) from MC38 cells expressing WT PD-L1 or the K136R mutant. (**C**) Immunoprecipitation (IP) and immunoblotting (IB) analysis of A375 cells with WT PD-L1 or K136R mutant interacting with purified HIS–PD-1. (**D** and **E**) Tumor growth curves of (**D**) NSG and (**E**) C57BL/6 mice inoculated with B16-F10 cells expressing PD-L1 WT or the K136R mutant (*n* = 5 per group). (**F**–**H**) CD8 and granzyme B (GZMB) immunostaining in the B16-F10 PD-L1 WT and PD-L1 K136R mutant tumors from C57BL/6 mice (*n* = 9); 3 tissue slides per tumor. (**F**) Representative images. Scale bar: 50 μm. (**G**) Quantification of CD8^+^ T cells. (**H**) Relative GZMB level. Unit = 262,144 μm^2^ (the area of the tumor tissue). **P < 0.01; ***P < 0.001, by unpaired, 2-tailed *t* test between 2 groups (**G** and **H**). Data are shown as the mean ± SEM.

K136, an evolutionarily conserved lysine located in the ECD domain, was identified as a K63-linked residue by mass spectrometry analysis (Supplemental Figure 8A). Upon mutation of K to R (termed K136R), PD-L1 displayed dramatically reduced ubiquitination by MIB2 (Figure 5A); however, its binding to MIB2 appeared unaffected (Supplemental Figure 8B). Human PD-L1 protein has 19 K residues, and we generated 17 K-to-R mutants (Supplemental Figure 8C). Ubiquitination analysis revealed that only the K136R mutant led to a drastic reduction of K63-linked ubiquitination by MIB2 (Supplemental Figure 8D). Consistently, the loss of MIB2-mediated PD-L1 K136 polyubiquitination decreased the membrane protein level of PD-L1 (Figure 5B) and impaired the interaction between PD-L1 and PD-1 protein (Figure 5C). Moreover, the PD-L1 K136R mutation did not affect B16-F10 tumor development in immunodeficient NSG mice (Figure 5D and Supplemental Figure 8E). In contrast, it slowed tumor growth in immunocompetent C57BL/6 mice (Figure 5E and Supplemental Figure 8F) and increased the number of tumor-infiltrating CD8^+^ T cells (Figure 5, F–H). In B16-F10 tumors from C57BL/6 mice, K136R mutation substantially reduced the endogenous K63-linked ubiquitination of PD-L1 (Supplemental Figure 8G). Collectively, these results reinforce that MIB2-mediated K63-linked polyubiquitination of PD-L1 impairs antitumor immunity.

### MIB2-mediated ubiquitination of PD-L1 drives its transportation to the plasma membrane via exocytosis.

**Figure 6:**
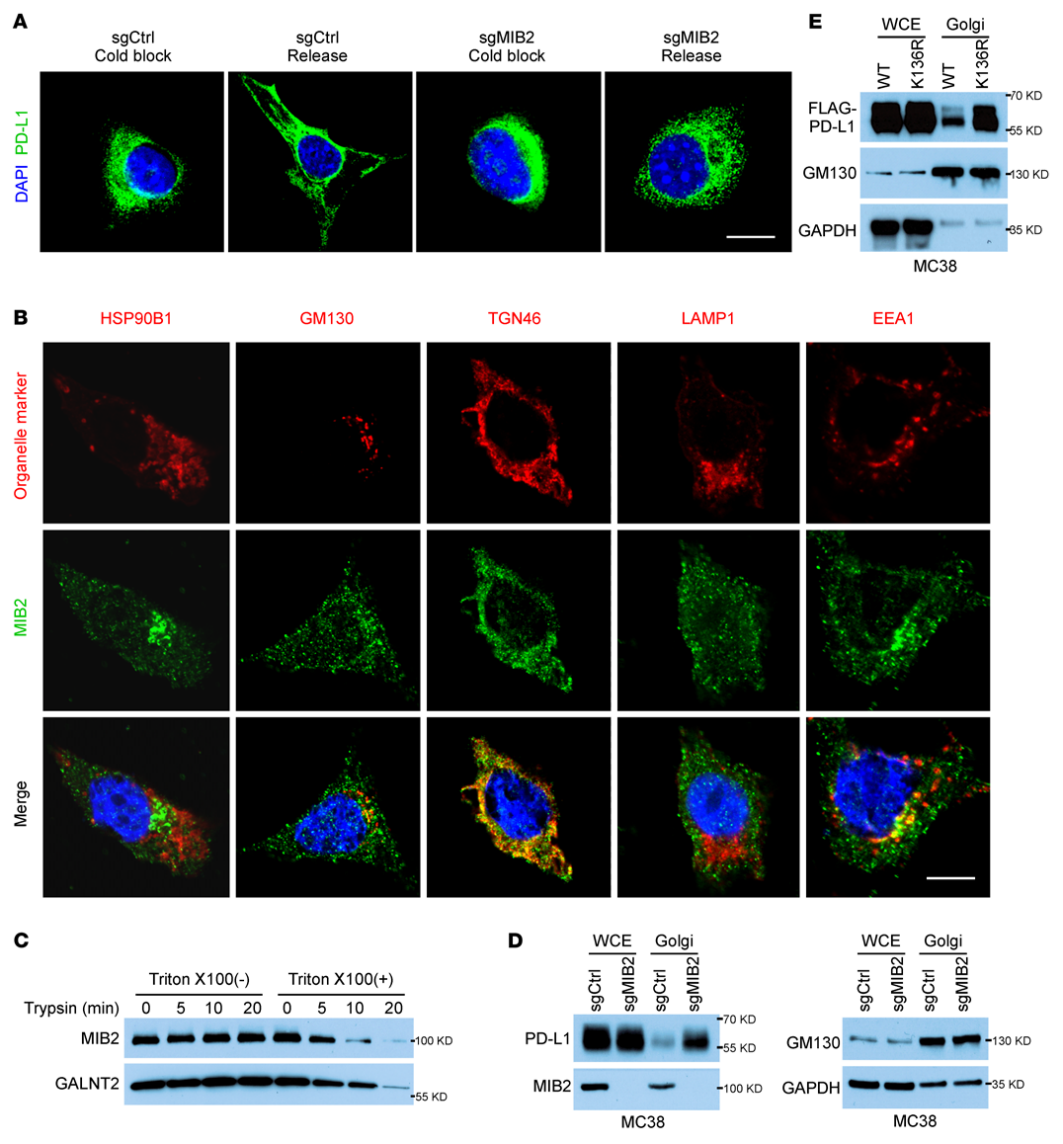
Ubiquitination by MIB2 is required for PD-L1 exocytosis. (**A**) Immunofluorescence analysis of PD-L1 in B16-F10 cells after cold block release. Scale bar: 10 μm. (**B**) Colocalization of MIB2 and subcellular organelles markers in B16-F10 cells. Scale bar: 10 μm. (**C**) Immunoblotting (IB) analysis of MIB2 and Galnt2 in trypsin-digested Golgi fractions with or without permeabilization. (**D**) IB analysis of PD-L1 in the whole-cell extract (WCE) and isolated Golgi from MC38 cells. (**E**) IB analysis of PD-L1 in the WCE and isolated Golgi from MC38 cells expressing PD-L1 WT or K136R mutant.

The amount of membrane proteins at the cell surface results from the balance of exocytosis, recycling, and endocytosis (41). As *MIB2* KO reduced the PD-L1 plasma membrane level, we speculated that MIB2 deletion either accelerates endocytosis or decreases exocytosis. A previous study reported that PD-L1–binding proteins were involved in endocytosis, nuclear transport, and export pathways (5). When endocytosis was blocked by MBCD or Pitstop 2, the percentage of increased membranous PD-L1 in *MIB2*-proficient B16-F10 cells was similar to that in *MIB2*-KO cells (Supplemental Figure 9, A and B), indicating that MIB2 deletion did not accelerate PD-L1 endocytosis. We then studied the effect of MIB2 depletion on the exocytosis pathway. Lowering the temperature of cells to 20°C allows endocytosis to continue but prevents protein exit from the TGN in the exocytosis arm (42–44). *MIB2*-proficient and *MIB2*-KO B16-F10 cells were incubated at 20°C before returning to 37°C (cold block release). IF data showed that PD-L1 stayed away from the plasma membrane under a cold block yet rapidly redistributed back to the plasma membrane after rewarming. However, the redistribution was not observed in *MIB2*-KO cells (Figure 6A). This result suggests that decreased PD-L1 on the plasma membrane in *MIB2*-KO cells is caused by the inhibition of exocytosis.

To understand how MIB2 may function to reduce PD-L1 exocytosis, we examined the subcellular localization of MIB2 by costaining of MIB2 with multiple subcellular organelles markers, including HSP90B1 (ER), GM130 (cis-Golgi), TGN46 (TGN), LAMP1 (lysosomal), and EEA1 (early endosomes). In addition to its presence in the early endosomal compartments (45), MIB2 was predominantly identified in the Golgi, where it colocalizes with TGN46 (Figure 6B). To further reveal the association of MIB2 in the Golgi, the Golgi fractions of B16-F10 cells were trypsin digested with or without prepermeabilization by Triton X-100. Trypsinization reduced the protein levels of MIB2 and a Golgi lumen protein GALNT2 in the permeable fractions. In contrast, signals for MIB2 and GALNT2 were unchanged in the nonpermeable fractions (Figure 6C). Subcellular fraction assay showed that the expression of PD-L1 was upregulated in the Golgi fraction in *MIB2*-KO MC38 cells (Figure 6D and Supplemental Figure 9C). Moreover, costaining PD-L1 with Golgi markers TGN46 and GM130 further revealed that depletion of MIB2 in MC38 cells caused an accumulation of PD-L1 in the TGN (Supplemental Figure 9, D and E). Reintroduction of MIB2 restored PD-L1 on the plasma membrane of MC38 cells (Supplemental Figure 9F). Mutation of the MIB2-catalyzed ubiquitination site K136 reduced PD-L1 membrane localization and increased PD-L1 expression in the Golgi fraction (Figure 6E and Supplemental Figure 9G). These findings indicate that PD-L1 ubiquitination by MIB2 in TGN is required for PD-L1 exocytosis from TGN to the plasma membrane.

**Figure 7:**
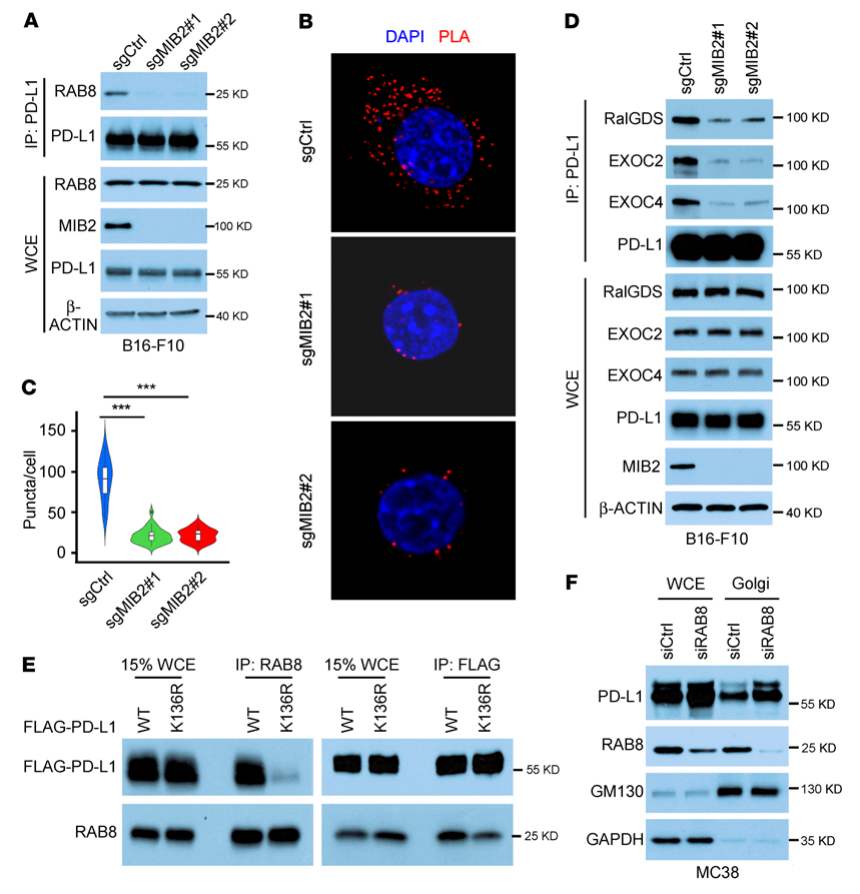
Ubiquitination by MIB2 is required for the PD-L1 and RAB8 interaction and exocytosis. (**A**) Coimmunoprecipitation (Co-IP) and immunoblotting (IB) analysis of endogenous PD-L1 and RAB8 in B16-F10 cells. (**B** and **C**) Proximity ligation assay (PLA) analysis of the PD-L1 and RAB8 interaction in MC38 cells. (**B**) Representative images. Scale bar: 10 μm. (**C**) Quantitation. Lines within the boxes denote median values; the tops of boxes represent the upper quartile (75th percentile), and bottoms of boxes represent the lower quartile (25th percentile); and widths denote cell densities. (**D**) Co-IP and IB analysis of endogenous PD-L1 and exocyst components in B16-F10 cells. (**E**) Co-IP analysis of the PD-L1 (WT or K136R) and RAB8 interaction in PD-L1–KO B16-F10 cells transfected with the indicated constructs. (**F**) IB analysis of PD-L1 in the whole-cell extract (WCE) and isolated Golgi from RAB8-silenced MC38 cells. ***P < 0.001, by 1-way ANOVA among 3 groups (**B** and **C**). Data are shown as the mean ± SEM.

To examine how MIB2 drives the ubiquitinated PD-L1 translocation from the TGN to the plasma membrane via exocytosis, we identified PD-L1–interacting proteins in 293T cells overexpressing MIB2 using mass spectrometry. Cellular organelles in the exocytic and endocytic pathways have a distinctive spatial distribution and communicate through an elaborate system of vesiculotubular transport. RAB proteins and their effectors coordinate consecutive stages of transportation, such as vesicle formation, vesicle and organelle motility, and tethering of vesicles to their target (46). Multiple RAB family proteins bound to PD-L1, and MIB2 overexpression enhanced their binding (Supplemental Figure 10A). Among the identified RABs, 4 RAB proteins (RAB8A, RAB10, RAB13, and RAB14) are functionally involved in protein trafficking from the TGN to the plasma membrane, RAB5 is involved in endocytosis, and RAB11 is involved in recycling (47). Co-IP revealed that the depletion of MIB2 reduces the interaction of PD-L1 with RAB8 but not with other identified RAB proteins in MC38 cells (Supplemental Figure 10B). The decreased interaction between PD-L1 and RAB8 after MIB2 deletion was also confirmed in B16-F10 and A375 cells (Figure 7A and Supplemental Figure 10C) (Figure 7A and Supplemental Figure 10, B and C). The PLAs confirmed that *MIB2* depletion impaired the interaction between PD-L1 and RAB8 in these cells (Figure 7, B and C, and Supplemental Figure 10D); reintroducing MIB2 rescued this interaction (Supplemental Figure 10E). RAB8 interacts with the exocyst, a highly conserved trafficking complex, to facilitate the targeting of newly synthesized proteins from the TGN to the plasma membrane (46–48). Costaining of RAB8 with TGN46 and exocyst components revealed that RAB8 colocalized with TGN46, EXOC2 (exocyst component), and RALGDS (exocyst activator; Supplemental Figure 10F). Co-IP revealed that the interaction between PD-L1 and exocyst components, including RALGDS, EXOC2, and EXOC4, was compromised in *MIB2*-KO cells (Figure 7D), indicating that *MIB2* KO inhibited exocytosis-mediated PD-L1 trafficking. PLAs for RAB8 and PD-L1 with antibodies specific for the ECD or the intracellular domain of PD-L1 revealed that the ECD but not the intracellular domain PLA exhibited strong Duolink signals (Supplemental Figure 10G). PLAs and co-IP showed that RAB8 preferred WT PD-L1 to the K136R mutant (Figure 7E and Supplemental Figure 10H). Moreover, RAB8 knockdown caused an upregulation of PD-L1 in the Golgi fraction (Figure 5F). These results suggest that the ubiquitinated PD-L1 exocytosis from the TGN to the plasma membrane is regulated by RAB8 and its downstream effectors, such as the exocyst.

### MIB2 positively correlates with membrane PD-L1 levels in non–small cell lung cancer.

To investigate the clinical relevance of our findings, we collected 93 non–small cell lung cancer (NSCLC) specimens for IHC analysis of MIB2 and PD-L1. Representative images of high and low MIB2 and PD-L1 membrane expression levels in tumor tissues are shown in Supplemental Figure 10A. We found that 54.8% of the tissues showed high MIB2 expression (Supplemental Figure 11A and Supplemental Table 2) and that MIB2 levels were positively correlated with PD-L1 membrane abundance (Supplemental Figure 11B). Of the 93 tumor specimens, 42 showed low MIB2 expression, 36 of which also showed low PD-L1 membrane staining. In contrast, 51 of the 93 specimens showed MIB2 overexpression, 28 of which also showed a strong PD-L1 membrane signal (Supplemental Figure 11, C and D, and Supplemental Table 2). We also examined MIB2 and PD-L1 mRNA levels related to NSCLC survival in published cohorts (*n* = 1,715 patients) (49). Higher MIB2 or PD-L1 mRNA levels were associated with worse overall survival, progression-free survival, and postprogression survival (Supplemental Figure 11, E and F).

**Figure 8:**
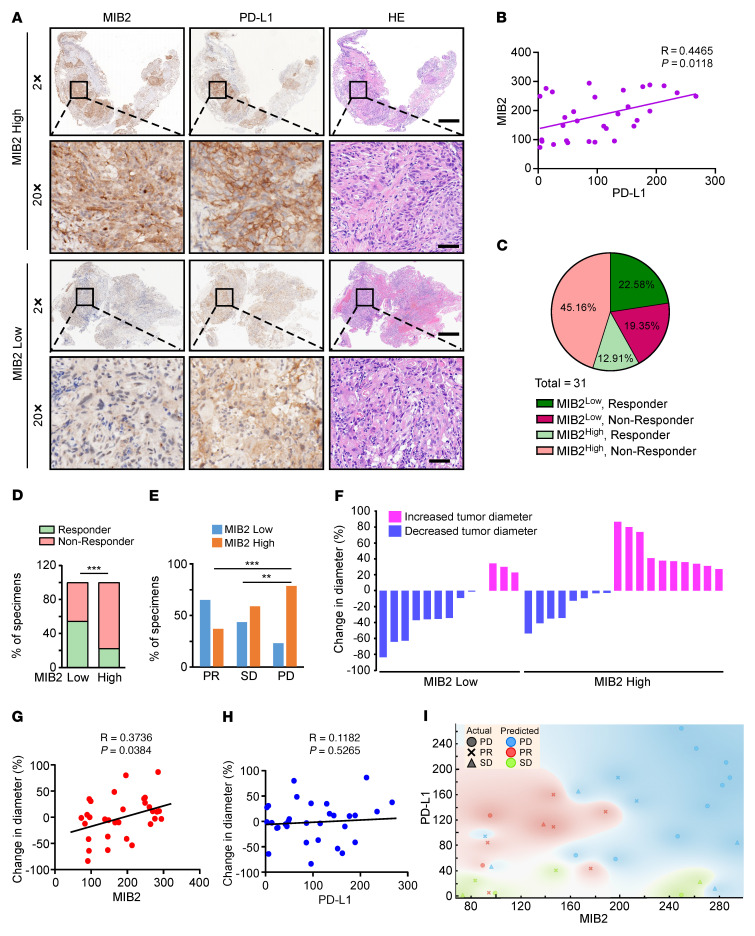
Membrane PD-L1 levels positively correlate with MIB2 expression in non–small cell lung cancer. (**A**) Representative IHC images for MIB2 and PD-L1 from nivolumab-treated patients with non–small cell lung cancer (NSCLC). The top two rows show images from a nonresponder; the bottom two rows show images from a responder. Scale bar: 500 μm (first and third rows); 50 μm (second and fourth rows). (**B**) Scatterplot showing the correlation between MIB2 and membrane PD-L1 levels in NSCLC specimens. Each plot represent 1 patient (*n* = 31). (**C**) Pie chart of MIB2 and membrane PD-L1 levels in 31 NSCLC specimens. (**D**) The percentage of responders and nonresponders displaying low or high MIB2 protein levels in 31 NSCLC specimens. (**E**) The percentages of patients with tumors exhibiting low or high MIB2 levels in the groups with partial response (PR), stable disease (SD), or progressive disease (PD). (**F**) Change in the diameter of tumors from patients with NSCLC treated with PD-1 mAb. Pink represents increased tumor diameter; and blue represents decreased tumor diameter. (**G** and **H**) Scatterplot showing the correlation between (**G**) MIB2 or (**H**) membrane PD-L1 levels and the response to PD-1 mAb treatment. (**I**) Naive Bayes model to classify 3 response groups based on the ratio of MIB2 and PD-L1 IHC scores. **P < 0.01; ***P < 0.001 by χ^2^ test for contingency (**D** and **E**).

The correlation between MIB2 and sensitivity to PD-1 mAb therapy was evaluated in a separate cohort of 31 patients with NSCLC who underwent needle biopsy before nivolumab treatment as first-line therapy, regardless of the PD-L1 status (Supplemental Table 3). Patients with partial response (PR) to PD-1 mAb were classified as responders, whereas patients with progressive disease (PD) or stable disease (SD) were classified as nonresponders (50). Representative IHC staining images of responders and nonresponders are shown in Figure 8A. There was a positive correlation between MIB2 expression and PD-L1 membrane abundance (Figure 8B). Of the 11 responders, 4 had tumors with high MIB2 expression; 14 of the 20 nonresponders had high MIB2 protein levels (Figure 8, C and D). The percentages of specimens with high or low MIB2 protein levels in the PR, SD, and PD groups are shown in Figure 8E. All 11 responders showed tumor shrinkage after 3 months of PD-1 mAb treatment, whereas 13 of the 20 nonresponders showed increased tumor size. The distribution of the percentage of change in tumor diameter and corresponding MIB2 protein levels in tumor specimens is summarized in Figure 8F. Notably, we found that the protein levels of MIB2 but not those of PD-L1 were positively correlated with changes in tumor size after 3 months of PD-1 mAb treatment (Figure 8, G and H). We used a naive Bayes model to evaluate whether the protein levels of MIB2 and/or membrane PD-L1 could be used to predict which patients may benefit from nivolumab. We found that the expression of both total MIB2 and membrane PD-L1 in the tumor epithelial region exhibited a predictive accuracy of 54.5% for PR (Figure 8I and Supplemental Figure 11G). However, the predictive accuracy of PR based on the expression of MIB2 or PD-L1 alone was 45.4% or 9.1%, respectively (Supplemental Figure 11G). Overall, these findings support that MIB2 is a potential predictive marker for PD-1 blockade therapy in NSCLC.

## Discussion

A common form of ICB resistance may arise from shuttling intracellular PD-L1 to the plasma membrane, as redistribution of PD-L1 to the plasma membrane impairs immunotherapy efficacy (51). The spatiotemporal distribution and dynamics at the cell surface of PD-L1 are strictly regulated in cancer cells. In this study, we identified MIB2 as an E3 ligase that facilitates the intracellular transportation of PD-L1 from the TGN to the plasma membrane through the exocytosis pathway. Unlike other E3 ligases, MIB2 does not reduce the total protein levels of PD-L1, nor does it appear to affect the intrinsic functions of PD-L1 in tumor cells (52). Instead, MIB2 adds K63-linked ubiquitination chains to PD-L1, which acts as a signal that enables its sorting from the Golgi to the plasma membrane. Exocytosis is an essential process that allows a cell to secrete molecules and peptides and regulate the composition of its plasma membrane. More than 60 RAB proteins, small monomeric GTPases of the Ras family, collectively regulate the flow of nearly all endomembrane traffic (46). Of these, RAB8 and RAB11 are found in the TGN, a major sorting station for secretory and membrane proteins (46). Previous studies have shown that RAB11 mediates endosomal recycling of PD-L1, prevents its redistribution to lysosomes for degradation, and restores PD-L1 levels at the plasma membrane (35, 36). We show that K63-linked ubiquitination mediates PD-L1 and RAB8 interaction. Unlike RAB11 in PD-L1 endosomal recycling, RAB8, as an intermediate, regulates PD-L1 membrane trafficking from the TGN to the plasma membrane by interacting with the ECD of PD-L1. With *MIB2* KO or RAB8 knockdown, PD-L1 is enriched in the Golgi. Thus, ubiquitination of PD-L1 by MIB2 is critical for its trafficking from the TGN to the plasma membrane through exocytosis.

We note that the migration pattern of all gel blots in this study showed PD-L1 at a weight of more than 55 kDa (a nascent PD-L1 peptide is of 33 kDa), suggestive of heavy glycosylation and other modifications in the ER and Golgi before MIB2-mediated ubiquitination. There are two possible models for MIB2 to catalyze PD-L1 ubiquitination within the Golgi. First, the ubiquitination process occurs inside the lumen of the TGN with some MIB2 and other needed proteins present. Second, the ECD of PD-L1 retrotranslocates across the TGN membrane towards the cytosol, is ubiquitinated by cytosolic MIB2, and translocates back into the lumen before shuttling to the plasma membrane. The second model has characteristics that partially resemble the ubiquitination of misfolded membrane proteins within the lumen of ER (53). Our experimental data from trypsin digestion with permeabilization and colocalization of PD-L1 and MIB2 with TGN46 favor the first model. However, we cannot completely rule out the second model, as MIB2 could be tightly associated with the outer membrane of Golgi (preventing trypsin digestion) and yet be stripped out upon treatment with Triton X-100.

PD-L1 expression is used to predict stratification of patients receiving PD-1 or PD-L1 mAb therapy (54); this process is complex because of variable antibodies and platforms, the subjective nature of scoring, and noninterchangeable definitions of PD-L1 positivity (55). Most companion or complementary diagnostic devices for PD-L1 expression levels in tumor cells used in approved ICB therapies or during clinical trials require membranous staining (56). As membrane PD-L1 levels are regulated by multiple factors (11, 57), knowledge of the regulation of the expression, stability, and trafficking of PD-L1 is crucial for optimizing patient stratification. We demonstrate a positive correlation between MIB2 expression and the membrane abundance of PD-L1 in tumor cells in NSCLC tissues. The combination of the total level of MIB2 and membrane PD-L1 or MIB2 only in NSCLC tumors is a better predictor than the level of membrane PD-L1 alone, should there be a need to select responders for anti–PD-1 (nivolumab) immunotherapy and assign nonresponders for clinical trials. The use of PD-L1 membranous staining to stratify patients for nivolumab treatment is no better than random assignment (~35.5%, 11 of 31), likely because PTMs such as N-glycosylation of PD-L1 compromise its detection in tumors (58). Without MIB2, PD-L1 within tumor cells cannot be translocated to the plasma membrane for its extrinsic function in binding PD-1 and evading immunity. Beyond PD-L1 ubiquitination, MIB2 may have other intrinsic oncogenic functions (59–61). MIB2-deficient tumors are more sensitive to PD-1 mAb in mice, making MIB2 a therapeutic target for enhancing immunotherapies. Thus, MIB2 is both a promising indicator for precision therapeutic stratification and an attractive target for improving the efficacy of ICB.

In summary, we revealed what we believe to be a new mechanism of immunosuppression in tumor cells through MIB2-mediated ubiquitination of PD-L1, which enables its translocation to the plasma membrane. With the assistance of RAB8, MIB2-mediated K63-linked ubiquitination chains act as a sorting signal required explicitly for PD-L1 protein translocation from the Golgi to the plasma membrane through exocytosis, the last step before PD-L1 performs its extrinsic immune-evading function. PD-L1 ubiquitination by MIB2 is a nonproteolytic PTM that regulates the spatial transportation of PD-L1 and antitumor immunity.

## Methods

Further information can be found in the Supplemental Methods.

### Cell lines.

A375, 293T, B16-F10, LLC1, and MC38 cell lines were cultured in DMEM, supplemented with 10% FBS. HCT116 and HT29 cells were maintained in McCoy’s 5A (modified) medium, supplemented with 10% FBS. A549 cells were cultured in RPMI-1640 medium supplemented with 10% FBS. All cell lines were obtained from ATCC and checked for mycoplasma.

### Mouse models.

The immunocompromised NSG and immunocompetent C57BL/6 mice (female, 6–8 weeks old) were obtained from The Jackson Laboratories. B16-F10 or MC38 tumors were established by subcutaneously injecting B16-F10 sgCtrl/sgMIB2 (1 × 10^5^) or MC38 sgCtrl/sgMIB2 (2 × 10^5^) cells in 100 μL PBS into the right flank of NSG/C57BL/6 mice. Tumors were measured by calipers every 2 days. Tumor volume was calculated using the following formula: tumor volume (mm^3^) = (length × width × width/2), where length was the longest diameter and width was the shortest diameter. Mice with tumors greater than 1,200 mm^3^ were sacrificed. For mouse PD-1 mAb treatment, MC38 sgCtrl/sgMIB2 (2 × 10^5^) cells in 100 μL of PBS were injected into the right flank of C57BL/6 mice. Seven days later, the mice were pooled and randomly divided into control antibody and PD-1 mAb (200 μg/mouse/100 μL PBS/every 3 days) treatment groups. Antibody administration was conducted by intraperitoneal injection every 3 days. The in vivo metastasis models were established by tail vein injection of B16-F10 sgCtrl/sgMIB2 (1× 10^5^) cells into NSG or C57BL/6 mice. For survival studies, animals were monitored with tumor volume measured every 2 days after tumor cell inoculation, and animals were euthanized when tumor volume exceeded 1,500 mm^3^.

### Plasmids.

FLAG–PD-L1 (RC213071) in the pCMV6-Entry vector and GFP–PD-L1 A (Del 1–18 AA; deletion of the amino acid residues 1–18), GFP–PD-L1 B (Del 19–238 AA), GFP–PD-L1 C (Del 239–259 AA), GFP–PD-L1 D (Del 260–290 AA), GFP–PD-L1 E (1–18 AA only), GFP–PD-L1 F (19–238 AA only), GFP–PD-L1 G (239–259 AA only), GFP–PD-L1 H (260–290 AA only), PD-L1 mutants (K25R, K41R, K46R, K75R, K89R, K105R, K124R, K129R, K136R, K162R, K178R, K185R, K263R, K270/271R, and K280/281R) were generated using the Q5 Site-Directed Mutagenesis kit (catalog E0554S, New England Biolabs) following the manufacturer’s standard protocol. All mutants were verified by DNA sequencing. FLAG-MIB2, HA-MIB2, and mutants, including MIB2 A (1–954 AA), MIB2 B (1–875 AA, Del RING domains), MIB2 C (1–507 AA), MIB2 D (125–999 AA), MIB2 E (175–999 AA), and MIB2 F (272–999 AA) were provided by Vanessa Redecke at St. Jude Children’s Research Hospital (Memphis, Tennessee, USA) (63). The customized E3 ligase shRNA library (137 E3 ligases, Supplemental Table 1), which contains 4 different shRNA oligonucleotides for each gene, was purchased from Sigma-Aldrich. The guaranteed sgRNAs targeting human/mouse MIB2 or PD-L1, and the shRNA targeting MIB2, were purchased from Horizon Discovery.

### Statistics.

All quantitative results are expressed as mean ± SEM of 3 independent experiments. Differences between means were evaluated by 2-tailed Student’s *t* test or ANOVA when data were normally distributed. The clinicopathologic significance of clinical samples was evaluated by the χ² test for categorical data. Kaplan-Meier analysis and the log-rank test (Mantel-Cox) were used for survival analysis. A *P* value of less than 0.05 was considered significant. The naive Bayes model was developed to predict treatment response for our NSCLC cohort using the open-source machine learning software Orange (64).

### Study approval.

All animal experiments were performed with the approval of the Institutional Animal Care and Use Committee of Baylor College of Medicine, following the guidelines established by the NIH Guide for the care and use of laboratory animals. For experiments using human samples, all samples were anonymously coded following local ethical guidelines (as stipulated by the Declaration of Helsinki). Written informed consent was obtained from patients, and the protocol was approved by the Ethical Review Board of Central South University.

## Author contributions

Xinfang Yu, WL, HL, and XW conceived and designed the study. Xinfang Yu, WL, HL, XW, C Coarfa, and Xinlian Yu acquired and/or analyzed and interpreted data. C Cheng, ZZ, and KHY provided additional conceptual advice, experimental design assistance, and technical support. YC coordinated the study sample collection. Xinfang Yu, WL, and YL drafted the manuscript. All authors revised the manuscript for important intellectual content and approved the final version submitted for publication.

## Supplementary Material

Supplemental data

## Version 1 02/01/2023

Electronic publication
