# Supplementary material for: PD-L1 translocation to the plasma membrane enables tumor immune evasion through MIB2 ubiquitination
Source: J Clin Invest. 2023 Feb 1;133(3):e160456. doi: 10.1172/JCI160456 (PMC9888393; doi:10.1172/JCI160456)
Supplement: Supplemental data [file jci-133-160456-s198.pdf]

Supplemental Data

Supplemental Methods.

Supplemental Figure S1. MIB2 regulates PD-L1 membrane levels in cancer cells.

Supplemental Figure S2. MIB2 KO compromised in vivo tumor development and metastasis in immunocompetent mice.

Supplemental Figure S3. Reintroduction of MIB2 rescued tumor growth in C57BL/6 mice.

Supplemental Figure S4. scRNA-seq analysis identifies tumor-infiltrating lymphocytes.

Supplemental Figure S5. Depletion of MIB2 improves the efficacy of anti-PD-L1 and anti-CTLA4 immunotherapies.

Supplemental Figure S6. MIB2 interacts with PD-L1.

Supplemental Figure S7. MIB2 phosphorylation promotes the E3 ligase activity.

Supplemental Figure S8. MIB2 promotes PD-L1 K136 ubiquitination.

Supplemental Figure S9. MIB2 is required for PD-L1 exocytosis.

Supplemental Figure S10. PD-L1 binds RAB8.

Supplemental Figure S11. PD-L1 membrane protein level positively correlates with MIB2 expression in NSCLC.

Supplemental Table S1: E3 ligase screened by shRNA knockdown

Supplemental Table S2: Protein expression of MIB2 and PD-L1 in 93 cases of NSCLC tissues.

Supplemental Table S 3 NSCLC patients with needle biopsy

Supplementary Methods

Clinical Tissue Collection. NSCLC was diagnosed and classified by the departments of Pathology of the Xiangya Hospital and the Third Xiangya Hospital of Central South University, following WHO guidelines. All specimens were collected following an Institutional Review Board-approved protocol. The needle biopsies of 31 NSCLC patients that responded or did not respond to Nivolumab (3 mg/kg, Q2W) were collected with written informed consent. All patient clinical information was summarized in Supplementary Table 3.

Cell Viability Assay. Cells were seeded at 2×10^3 cells/well in 96-well plates and maintained for 72 h. The reagent MTS (Promega) was added to measure cell viability following the manufacturer's standard protocol.

Immunoblot (IB) and Co-immunoprecipitation (co-IP) Analysis. Whole-cell lysates were prepared with RIPA buffer ((20 mM NAP, pH7.4, 150 mM NaCl, 1% Triton, 0.5% Sodium-deoxycholate, and 0.1% SDS) containing protease inhibitor. The lysates were sonicated and centrifuged at 12,000 × g for 15 min at 4°C and subjected to protein concentration using BCA Assay Reagent (Thermo Fisher Scientific). The proteins were denatured and resolved by SDS–PAGE and immunoblotted with primary/second antibodies as follows: PD-L1 (13684, 1:1,000; Cell Signaling Technology), PD-L1 (29122, 1:1,000; Cell Signaling Technology), TRIM3 (ab111840, 1:1,000, Abcam), BARD1 (ab50984, 1:1,000, Abcam), FBW7 (40-1500, 1:1,000; Thermo Fisher Scientific), RBX1 (11922, 1:1,000; Cell Signaling Technology), MIB2 (ab233068, 1:1,000; Abcam), MIB2 (ab99378, 1:1,000; Abcam), MIB2 (MAB72891, 1:500; R&D system), GALNT2 (pa5-21541, 1:1000; Thermo Fisher Scientific), β-ACTIN (A2228, 1:10,000; Sigma-Aldrich), α-TUBULIN (T5168, 1:5,000; Sigma-Aldrich), Na/K ATPase (ab7671, 1:2,000; Abcam), GFP-Tag (TA150032, 1:5000; Origene), FLAG-Tag (14793, 1:5000; Cell Signaling Technology), FLAG-HRP (A8592, 1:10,000; Sigma-Aldrich), HA-Tag (3724, 1:5000; Cell Signaling Technology), HIS-Tag (A00186, 1:4000; GenScript), GST-Tag (A190-122A, 1:4000; Bethyl Lab), K63-linkage specific polyubiquitin eabbit mAb (12930, 1:1000; Cell Signaling Technology), RAB8A (ab188574, 1:1000; Abcam), RAB8B (ab222017, 1:1000; Abcam), RAB8 (6975, 1:1000; Cell Signaling Technology), RAB8 (610845, 1:1000; BD Biosciences), RAB10 (8127, 1:1000; Cell Signaling Technology), RAB11B (ab3612, 1:1000; Abcam), RAB13 (PA5-13621, 1:1000; Thermo Fisher Scientific), RAB14 (PA5-

55306, 1:1000; Thermo Fisher Scientific), GM130 (610823, 1:1000; BD Biosciences), and GAPDH (2118, 1:4000; Cell Signaling Technology). For co-IP assays, cell lysates were prepared using IP Lysis Buffer supplemented with protease inhibitor. Add primary antibody (2 μg) and protein A/G agarose beads (40 μL, 50% slurry) to 500 μg cell lysate, and incubate with rotation overnight at 4°C. The agarose beads bound with target proteins were washed with ice-cold PBS and eluted with 2×loading buffer. Protein interaction was examined by immunoblotting.

In vitro Ubiquitination Assay. HA-MIB2 and HA-MIB2 Del RING protein were expressed in 293T cells, immunoprecipitated with anti-HA magnetic beads, and eluted with the HA peptide. HA-MIB2 and HA-MIB2 Del RING protein along with GST-PD-L1 protein (Proteintech) were incubated with 40 nM UbE1 (E1), 0.7 μM UbcH5a (E2), and 10 μM ubiquitin for 4 h at 37°C in a reaction buffer (40 mM Tris-HCl, pH 7.5, 10 mM $MgCl_2$, 0.6 mM DTT, and 2 mM ATP). Reactions were stopped by adding 500 μL RIPA buffer supplemented with protease inhibitor and 10 mM N-ethylmaleimide (NEM) before being immunoprecipitated with PD-L1 antibody overnight at 4°C. PD-L1 ubiquitination was examined by IB.

T Cell-mediated Tumor Cell Killing Assay. T cell-mediated tumor cell killing was performed as described previously (31). Briefly, human peripheral blood mononuclear cells (PBMC) were purchased from STEMCELL Technologies (Vancouver, BC, Canada) and cultured in ImmunoCult-XF T cell expansion medium (10981; STEMCELL Technologies). ImmunoCult Human CD3/CD28/CD2 T cell activator (10970; STEMCELL Technologies) and IL-2 (10 ng/mL; PeproTech, Rocky Hill, NJ, USA) were added to the culture medium and maintained for one week following the manufacturer's protocols. The activated T cells were maintained in DMEM/F12 medium supplied with anti-CD3 antibody (100 ng/mL; 16-0037; Thermo Fisher Scientific) and IL-2 (10 ng/mL) co-cultured with A375 stable cells for 48 h. After extensive washing with PBS, the living tumor cells were quantified by a spectrometer at OD (570 nm) followed by crystal violet staining.

Subcellular Fraction Isolation. Proteins from different cellular compartments were prepared using a Subcellular Protein Fractionation Kit (Thermo Fisher Scientific). The Golgi Apparatus Enrichment Kit (Invent Biotechnologies) was used for Golgi apparatus isolation.

Nickel Beads Pulldown Assay. The Nickel beads pulldown assay for ubiquitination analysis was performed as described previously (63). Briefly, the cells were lysed with a lysis buffer (6 M guanidine-HCl, 0.1 M Na_2HPO_4/NaH_2PO_4, 0.01 M Tris/HCl, pH 8.0, 5 mM imidazole, and 10 mM β-mercaptoethanol [ME]), supplemented with protease inhibitors and 10 mM NEM. Cell lysates were sonicated and centrifuged at 13,000 × g for 10 min. Add 40 μL Ni-NTA-agarose beads (Qiagen Inc) to the supernatant and incubate with rotation for four h at room temperature. The mixture was centrifuged at 5,000 × *g* for 5 min at room temperature. Resuspend the pellet and washed sequentially with the following buffers: (**A**) 6 M guanidine-HCl, 0.1 M Na_2HPO_4/NaH_2PO_4, 0.01 M Tris/HCl, pH 8.0, 5 mM imidazole plus 10 mM β-ME; **(B)** 8 M Urea, 0.1 M Na_2HPO_4/NaH_2PO_4, 0.01 M Tris/HCl, pH 8.0, 10 mM imidazole, 10 mM β-ME plus 0.1% Triton X-100; **(C)** 8 M urea, 0.1 M Na_2HPO_4/NaH_2PO_4, 0.01 M Tris/HCl, pH 6.3, 10 mM β-ME, 20 mM imidazole plus 0.2% Triton X-100; **(D)** 8 M urea, 0.1 M Na_2HPO_4/NaH_2PO_4, 0.01 M Tris/HCl, pH 6.3, 10 mM β-ME, 10 mM imidazole plus 0.1% Triton X-100; and **(E)** 8 M urea, 0.1 M Na_2HPO_4/NaH_2PO_4, 0.01 M Tris/HCl, pH 6.3, 10 mM β-ME, 10 mM imidazole plus 0.05% Triton X-100. The beads were boiled with 2× SDS sample-loading buffer containing 200 mM imidazole and subjected to IB analysis.

Generation of Stable Cell Lines. The plasmids containing control sgRNA (Cat. GSGC11963), or sgRNAs targeting MIB2 (For human MIB2 gene, sgMIB2#1: Cat. GSGH11935-247682429; sgMIB2#2: Cat. GSGH11935-247645005. For mouse MIB2 gene, sgMIB2#1: Cat. GSGM11941-247900450; sgMIB2#2: Cat. GSGM11941-247852972.)/PD-L1 (For human PD-L1 gene, sgPD-L1: Cat. GSGH11935-247641760. For mouse PD-L1 gene, sgPD-L1: Cat. GSGM11941-247819534) were purchased from Horizon Discovery (Waterbeach, UK). To generate CRISPR-Cas9-based *MIB2* and *PD-L1* KO stable cells, the sgRNA were transiently transfected into the cells for 48 h, puromycin (2 μg/ml) was added into the cell culture medium and maintained for 2-3 weeks. Individual colonies were picked up, and the KO efficacy was examined by IB. To generate *MIB2*-knockdown B16-F10 stable cells, shMIB2 (Cat. TRCN0000041148, TRCN0000041150) were co-transfected into 293T cells with psPAX2 and pMD2.G, the viral supernatant fractions were collected at 72 h after transfection, and followed by infection into cells together with 8 μg/mL polybrene. Puromycin selection was initiated at 48 h after infection and maintained for 1-2 weeks. The positive colonies with MIB2 knockdown were verified by immunoblotting analysis.

Immunofluorescence (IF). For cell staining, tumor cells were seeded into an μ-Slide (ibidi) for 24 h, fixed in 4% paraformaldehyde (Santa Cruz) for 10 min, and permeabilized in 0.2% Triton X-100 (Thermo Fisher Scientific) for 15 min at room temperature. Cells were washed with ice-cold PBS and incubated with BSA blocking buffer for 1 h. Add specific primary antibodies into the slides and incubate at 4°C in a humidified chamber overnight. After being washed with ice-cold PBS, the slides were incubated with fluorescent-dye conjugated secondary antibodies for 30 min at room temperature. DAPI (ab104139, Abcam) was used for nuclei counterstaining. The following antibodies were used for cell staining: PD-L1 ECD (86744, 1:200; Cell Signaling Technology), PD-L1 (13684, 1:200; Cell Signaling Technology), GM130 (610823, 1:200; BD Biosciences), TGN46 (MA3-063, 1:200; Thermo Fisher Scientific), HSP90B1 (4877, 1:200; Cell Signaling Technology), EEA1 (ab2900, 1:200; Abcam), EXOC2 (NBP3-04535, 1:200; Novus Biologicals), RalGDS (PA5-116790, 1:200; Fisher Scientific-Thermo Scientific). For tissue immunofluorescence, paraffin tumor tissue sections from syngeneic mouse model or NSCLC patient samples were baked at 60°C for 2 h, followed by deparaffinization and rehydration in a series of graded alcohols until water was used. Antigen retrieval was performed by submerging the tissue samples into boiling sodium citrate buffer (10 mM, pH 9.0), maintaining for 10 min, and incubating with 3% H_2O_2 in methanol for another 10 min. Tissue samples were blocked with BSA Blocking Buffer for 1 h at room temperature. Primary antibodies targeting CD8A (#14-0081-82, 1:100, Thermo Fisher Scientific), Granzyme B (ab255598, 1:100, Abcam), and PD-L1 (ab213480, 1:100, Abcam) were added to the tissue slides and incubated overnight at 4°C in a humidified chamber. After extensive washing with PBS, fluorescent-dye conjugated secondary antibodies were added to the tissue slides and incubated for 45 min at room temperature. DAPI was used for nuclei counterstaining. Images were captured on a Leica SP8 Confocal microscope. For live cells binding to PD-1-Fc, Recombinant Human PD-1 Fc Chimera Alexa Fluor® 488 Protein was used (R&D Systems).

Proximity-Ligation Assay (PLA). Cells were seeded into an 18 well μ-Slide and incubated for 24 h. After fixing with 4% paraformaldehyde at room temperature for 10 minutes, the cells were permeabilized with ice-cold methanol for 15 min. The washed μ-Slide was treated by the Duolink Blocking buffer for 30 min at room temperature, followed by primary antibodies overnight at 4 °C, and the secondary antibodies for 1 h at 37°C. The slides were washed in PBS with 0.5% Tween-20 for 10min. Ligation and amplification steps were conducted according to the manufacturer's

instructions. Mounting media with DAPI (Abcam) was used for Nuclei counterstaining, and images were acquired on a Leica SP8 Confocal microscope. Puncta were determined by PLA spots counting in each cell. Violin graph with box and whisker plots display the median with whiskers incorporating 10-90% of all data, and outliers were displayed as dots. To determine the binding between MIB2 and PD-L1, primary antibodies for PD-L1 (13684, 1:200; Cell Signaling Technology), MIB2 (MAB72891, 1:200; R&D Systems), PD-L1 (ab269674, 1:200; Abcam), and MIB2 (ab233068, 1:200; Abcam) were applied to duo-link assay (DUO92101, Sigma-Aldrich). To show the interaction between RAB8 and PD-L1, primary antibodies for PD-L1 (13684, 1:200; Cell Signaling Technology), RAB8 (610845, 1:200; BD), and PD-L1 (ab213480, 1:200; Abcam) were used in the PLA assays.

Immunohistochemical (IHC) staining. The tissue sections were baked at 60°C for 2 h, deparaffinized in xylene, and rehydrated with decreasing ethanol concentrations in water. Slides were submerged into boiling sodium citrate buffer (10 mM, pH 9.0) for 10 min and incubated with 3% H_2O_2 in methanol for 10 min. The tissue samples were blocked with BSA Blocking Buffer for 1 h at room temperature. Primary antibodies targeting MIB2 (#PA5-66985, 1:400, Thermo Fisher Scientific) and PD-L1 (#13684, 1:100, Cell Signaling Technology) were added to the tissue slides and incubated overnight at 4°C in a humidified chamber. After extensive washing with PBS, fluorescent-dye conjugated secondary antibodies were added to the tissue sample and incubated for 45 min at room temperature. The target proteins were visualized by diaminobenzidine (DAB) substrate, and hematoxylin was used for nuclei counterstaining. IHC was scored numerically for both percentages (ranging from 0 to 100%) of positive cells and intensity (ranging from 0 to 3. 0, no staining; 1, weak staining; 2, intermediate staining; 3, strong staining). IHC score = [1 × (% of cell with weak intensity) + 2 × (% of cell with intermediate intensity) + 3 × (% of cell with strong intensity)] × 100. An IHC score of less than 150% was considered a low expression, whereas a score greater than 150% was considered a high expression.

Mass spectrometry (MS) analysis. To discover PD-L1-binding proteins, 293T cells were transfected with FLAG-PD-L1 only or co-transfected with FLAG-PD-L1 and HA-MIB2 for 48 h. Cells were lysed in the IP buffer containing protease inhibitors. IP was performed using the FLAG-

tag antibody (Cat. #F1804, Sigma-Aldrich) at 4°C overnight. The immunoprecipitated proteins were resolved by sodium dodecyl sulfate-polyacrylamide gel electrophoresis (SDS-PAGE), followed by Coomassie blue staining (Cat. #BP101-25, Thermo Fisher). To identify the ubiquitination sites of PD-L1, FLAG-PD-L1 and His-Ub were co-transfected with/without MIB2 into 293T cells for 72 h. Cell lysates were prepared with modified RIPA buffer (20 mM NAP, pH7.4, 150 mM NaCl, 1% Triton, 0.5% Sodium-deoxycholate, and 1% SDS) containing protease inhibitor and 10 mM N-Ethylmaleimide (NEM). The lysates were sonicated and boiled at 95°C for 5 min, diluted with 0.1% SDS contained RIPA buffer, and centrifuged at 12,000 × g for 15 min at 4°C. The supernatant was subjected to IP analysis using the FLAG-tag antibody at 4°C overnight. The immunoprecipitated proteins were resolved by sodium dodecyl sulfate-polyacrylamide gel electrophoresis (SDS-PAGE). The peptides were analyzed using all collected collision-induced dissociation spectra and the search programs Sequest and Mascot. Protein and peptide identifications were validated using Scaffold.

scRNA-seq.

Cell preparation and sequencing. Pooled fresh tumors were collected from three mice in shCtrl, shMIB2#1, and shMIB2#2 each group. Enzyme mix was prepared by adding 2.35 mL of RPMI 1640, 100 μL of Enzyme D, 50 μL of Enzyme R, and 12.5 μL of Enzyme A into a gentleMACS C Tube. Tumor tissues were cut into small pieces of 2–4 mm. Single-cell suspensions were prepared using Tumor Dissociation Kit (130-096-730, Miltenyi Biotec) by Miltenyi Biotec gentleMACS Octo Dissociator (130-096-427, Mitenyi Biotec). Red Blood Cell Lysis Solution (10×) (# 130-094-183) was used to remove erythrocytes or dead cells. The mixtures were resuspended into single cells with 0.4% BSA for 10x genomics processing. Library was performed according to the manufacturer's instructions (Chromium Single Cell V(D)J Reagent Kits (v1.1 Chemistry), 10x Genomics). The final libraries contain the P5 and P7 priming sites were used in Illumina sequencing.

Libraries were run on Illumina NextSeq 500.

Primary analysis of raw reads. Raw reads were processed with fastQC and fastp to remove low-quality reads. Poly-A tails and adaptor sequences were removed by cutadapt. After quality control, reads were mapped to the reference genome GRCh38 (Ensembl version 92 annotation) using STAR. Gene counts and UMI counts were acquired by featureCounts software. Expression matrix files for subsequent analyses were generated based on gene counts and UMI counts.

Quality control, dimension-reduction, and clustering. Before analyses, cells were filtered by UMI counts below 30,000 and gene counts between 200 to 5,000, followed by removing the cells with over 20% mitochondrial content. After filtering, the functions from Seurat v2.3 (66) were used for dimension reduction and clustering. We then used NormalizeData and ScaleData functions to normalize and scale all gene expressions and selected the top 2000 variable genes with the FindVariableFeautres function for PCA analysis. Using the top 20 principal components, we separated cells into multiple clusters with FindClusters. The batch effect between samples was removed by Harmony (67). Finally, the UMAP algorithm was applied to visualize cells in a two-dimensional space.

Differentially expressed genes (DEGs) analysis. Genes expressed in more than 10% of the cells in a cluster and with an average log (Fold Change) of greater than 0.25 were selected as DEGs by Seurat v3.1.2 FindMarkers based on Wilcox likelihood-ratio test with default parameters.

Cell type annotation. The cell-type identity of each cluster was determined with the expression of canonical markers found in the DEGs using the SynEcoSys database. Heatmaps displaying the expression of markers used to identify each cell type were generated by Seurat v3.1.2 DoHeatmap/DotPlot/Vlnplot. scRNA-seq data analyses were assisted by Singleron Biotechnologies (Nanjing, China).

Data repository. scRNA-seq data were deposited into the NCBI Gene Expression Omnibus with an accession number GSE218906.

Supplemental Figure 1

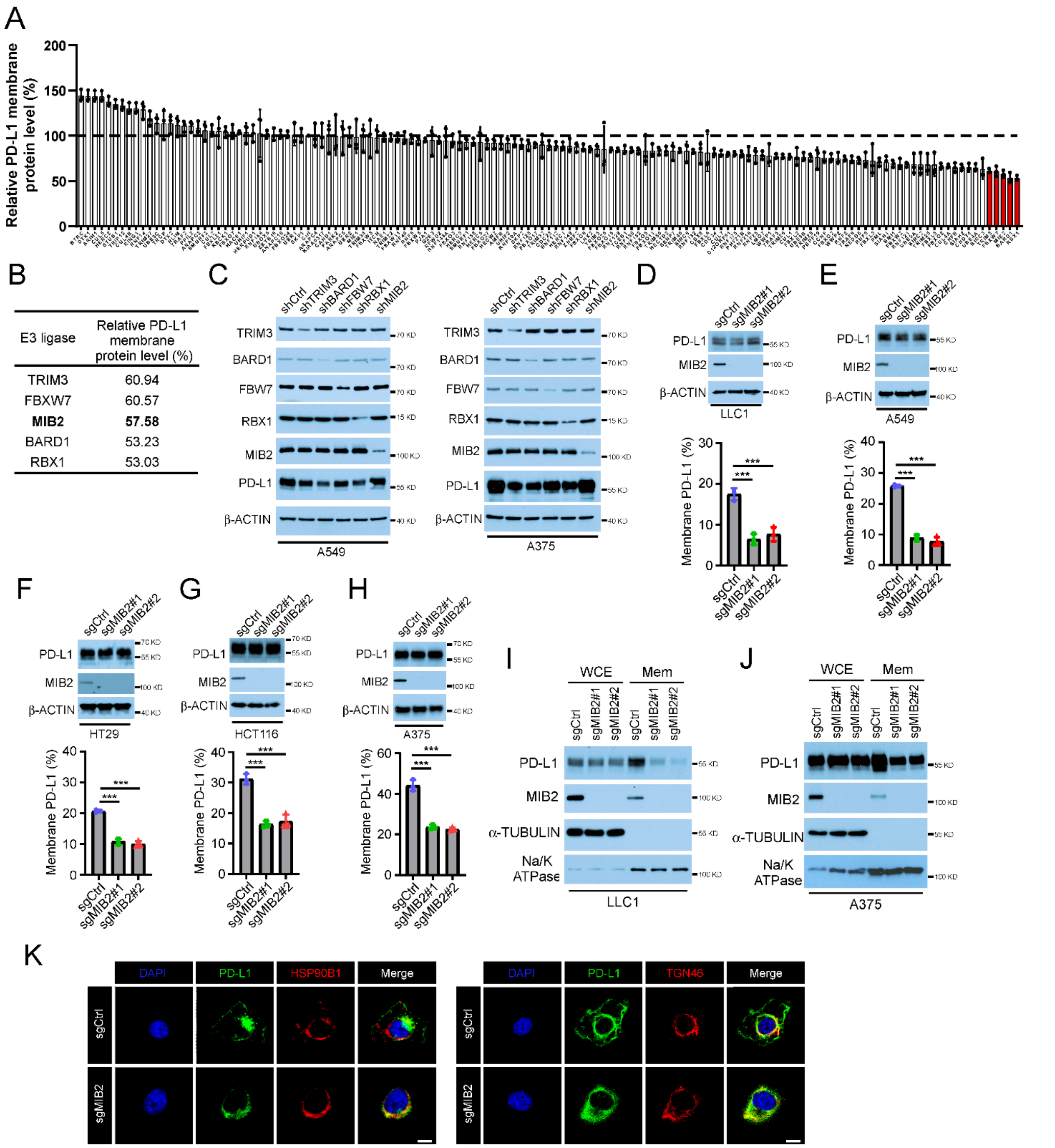

Figure S1. MIB2 regulates PD-L1 membrane levels in cancer cells. A, Quantification of PD-L1 membrane protein levels upon knockdown of each E3 ligase in A375 cells. A total of 137 shRNA pools were individually transfected into A375 cells, PD-L1 membrane protein levels were determined by fluorescence-activated cell sorting (FACS) analysis after 48 h. **B**, List of top 5 candidate E3 ligases whose knockdown led to reduced PD-L1 membrane abundance. **C**, A549 and A375 cells were transfected with shCtrl or shRNA targeting one E3 ligase, and target proteins were examined by immunoblotting (IB) analysis as indicated. **D-H**, IB (top) and FACS (bottom) analysis of PD-L1 protein levels in LLC1 (**D**), A549 (**E**), HT29 (**F**), HCT116 (**G**), and A375 (**H**) cells with sgCtrl or sgMIB2. **I, J**, IB analysis of PD-L1 protein levels in whole-cell extract (WCE) and membrane fractions (Mem) from LLC1 (**I**) and A375 (**J**) sgCtrl/sgMIB2 stable cells. **K**, A375 cells co-stained with antibodies against PD-L1 and ER (HSP90B1) and Golgi markers (TNG46). ***, $p < 0.001$, by 1-way ANOVA among 3 groups (D-H). Data indicate the mean ± SD.

Supplemental Figure 2

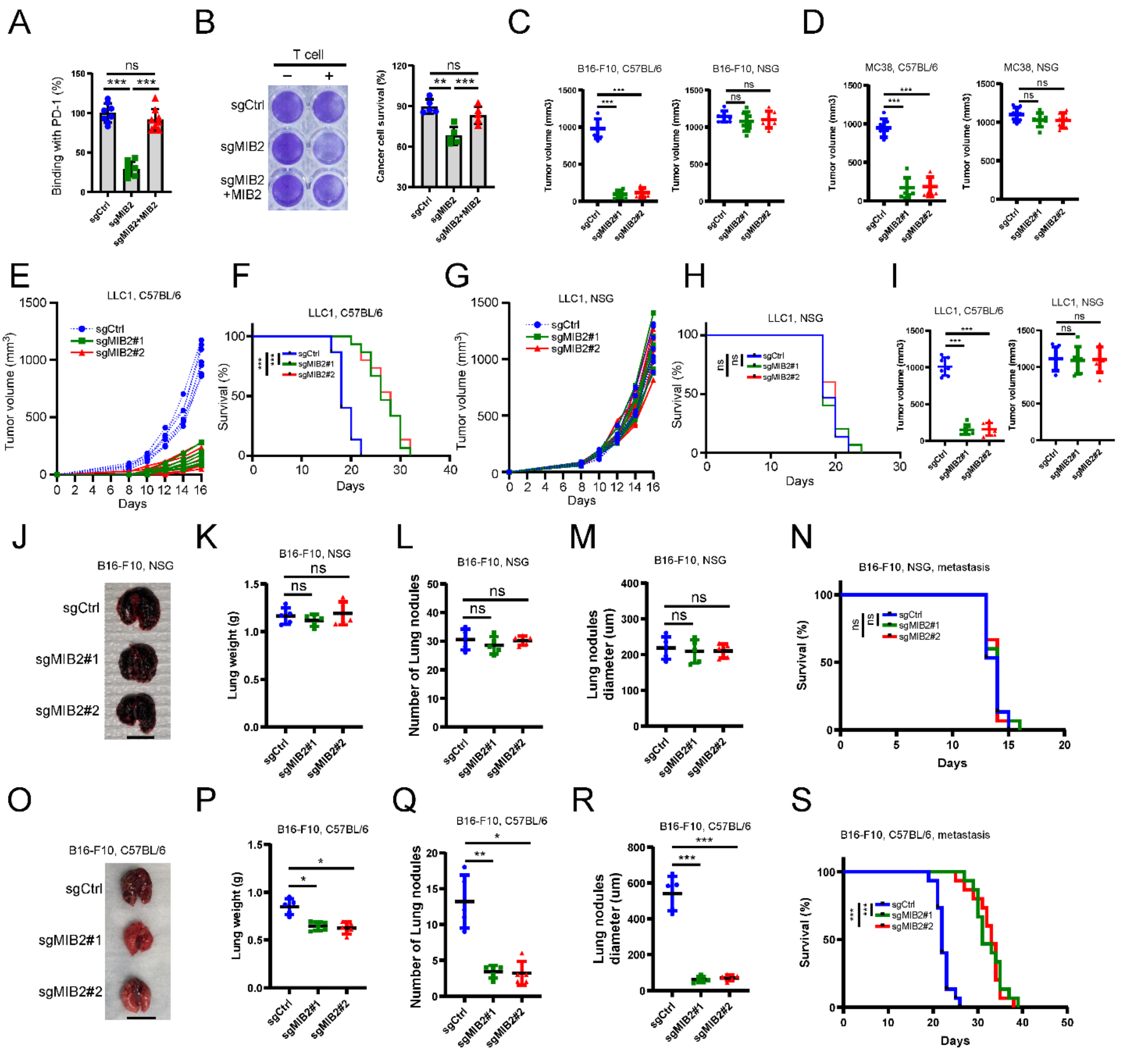

Figure S2. MIB2 KO compromised in vivo tumor development and metastasis in immunocompetent mice. A, The effect of MIB2 reintroduction into MIB2 KO A375 cells on PD-L1 binding to green-fluorescent-labeled PD-1-Fc. Data represented mean ± SD (n = 7). **B**, A375 cell survival under T cell killing. A375 cells were co-cultured with or without activated T cells (with a ratio of 1:5) for 48 h and were subjected to crystal violet staining. **C**, Volumes of B16-F10 (sgCtrl or sgMIB2) tumors in C57BL/6 and NSG mice. **D**, Volumes of MC38 (sgCtrl or sgMIB2) tumors in C57BL/6 and NSG mice. **E**, Growth curve of LLC1 tumors in C57BL/6 mice (n = 7 per group). **F**, Kaplan–Meier survival curves of LLC1 tumor-bearing C57BL/6 mice (n = 15 per group). ***, p < 0.001. **G**, Growth curve of LLC1 tumors in NSG mice (n = 7 per group). **H**, Kaplan–Meier survival curves of LLC1 tumor-bearing NSG mice. ns, not statistically significant (n = 15 per group). **I**, Volumes of LLC1 (sgCtrl or sgMIB2) tumors in C57BL/6 and NSG mice. **J-S**, B16-F10 (sgCtrl and sgMIB2) metastasis. Tumor cells were injected into NSG (**J-N**) or C57BL/6 (**O-S**) mice via tail vein, and after 12 days, animal lungs were analyzed (**J-N** & **O-S**): representative images of lung metastasis (**J, O**); lung weight (**K, P**); and the number (**L, Q**) and the diameter (**M, R**) of lung metastasis nodules. Scale bar, 1 cm. Kaplan-Meier survival curves for separate groups of animals injected with B16-F10 cells via tail vein (**N**,**S**) (n = 15 per group). *, p<0.05; **, p<0.01; ***, p<0.001, by log-rank (Mantel-Cox) test (F, H, N, and S); and by 1-way ANOVA among 3 groups. ns, not statistically significant. Data indicate the mean ± SD.

Supplemental Figure 3

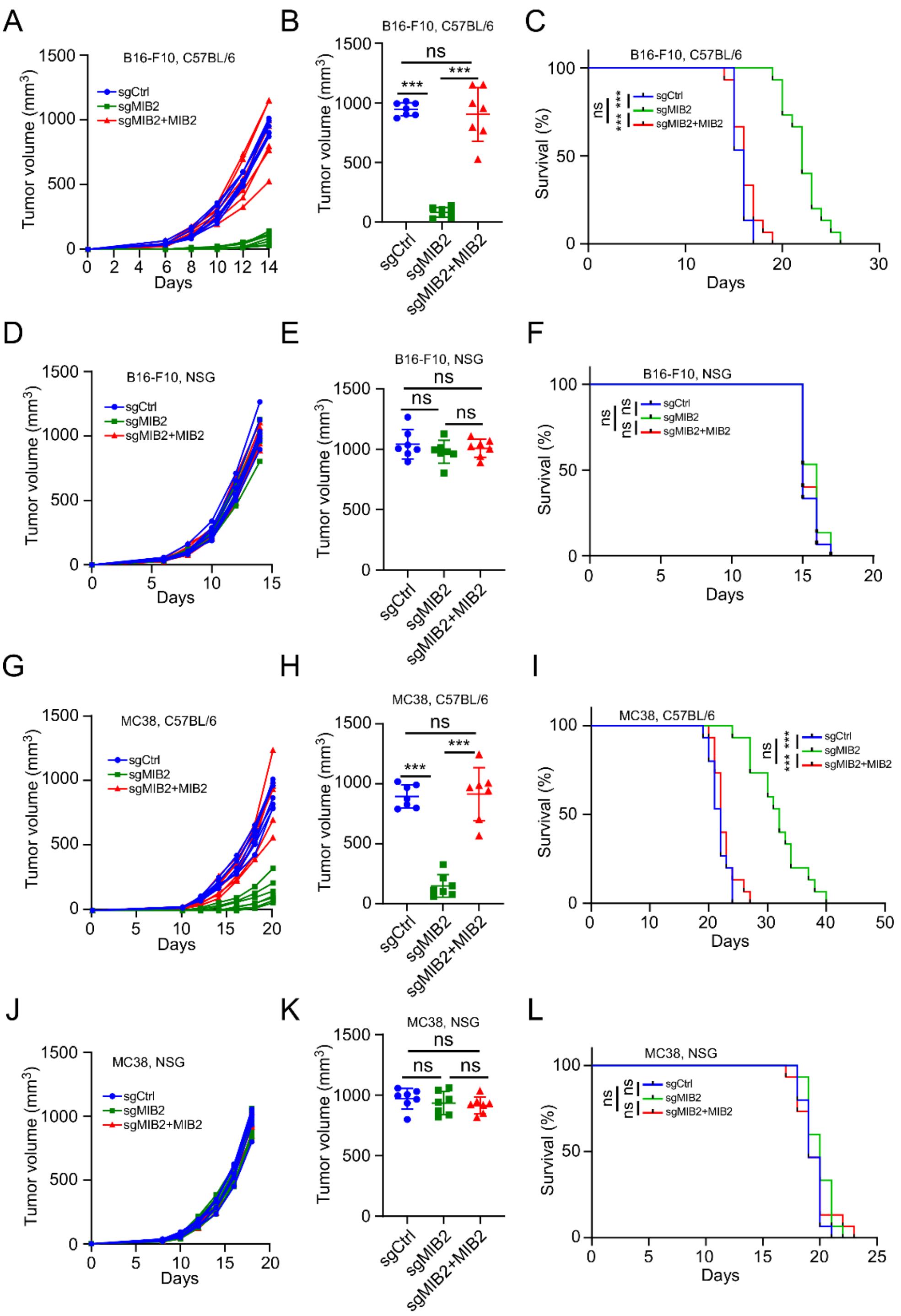

Figure S3. Reintroduction of MIB2 rescued tumor growth in C57BL/6 mice. A,B, The growth curve (**A**) and average tumor volume (**B**) of B16-F10 (sgCtrl, sgMIB2, or MIB2 reintroduced) tumors in C57BL/6 mice (n = 7 per group). **C**, Kaplan-Meier survival curves of B16-F10 tumor-bearing C57BL/6 mice from **A** (n = 15 per group). **D,E**, The growth curve (**D**) and average tumor volume (**E**) of B16-F10 tumors in NSG mice (n = 7 per group). **F**, Kaplan-Meier survival curves of B16-F10 tumor-bearing NSG mice from **D** (n = 15 per group). **G,H**, The growth curve (**G**) and average tumor volume (**H**) of MC38 (sgCtrl, sgMIB2, or MIB2 reintroduced) tumors in C57BL/6 mice (n = 7 per group). **I**, Kaplan-Meier survival curves of MC38 tumor-bearing C57BL/6 mice from **G** (n = 15 per group). **J,K,** The growth curve (**J**) and average tumor volume (**K**) of MC38 tumors in NSG mice. ns, not statistically significant (n = 7 per group). **L**, Kaplan-Meier survival curves of MC38 tumor-bearing NSG mice from **J** (n = 15 per group). ***, p<0.001, by log-rank (Mantel-Cox) test (C, F, I, and L); and by 1-way ANOVA among 3 groups. ns, not statistically significant. Data indicate the mean ± SEM.

Supplemental Figure 4

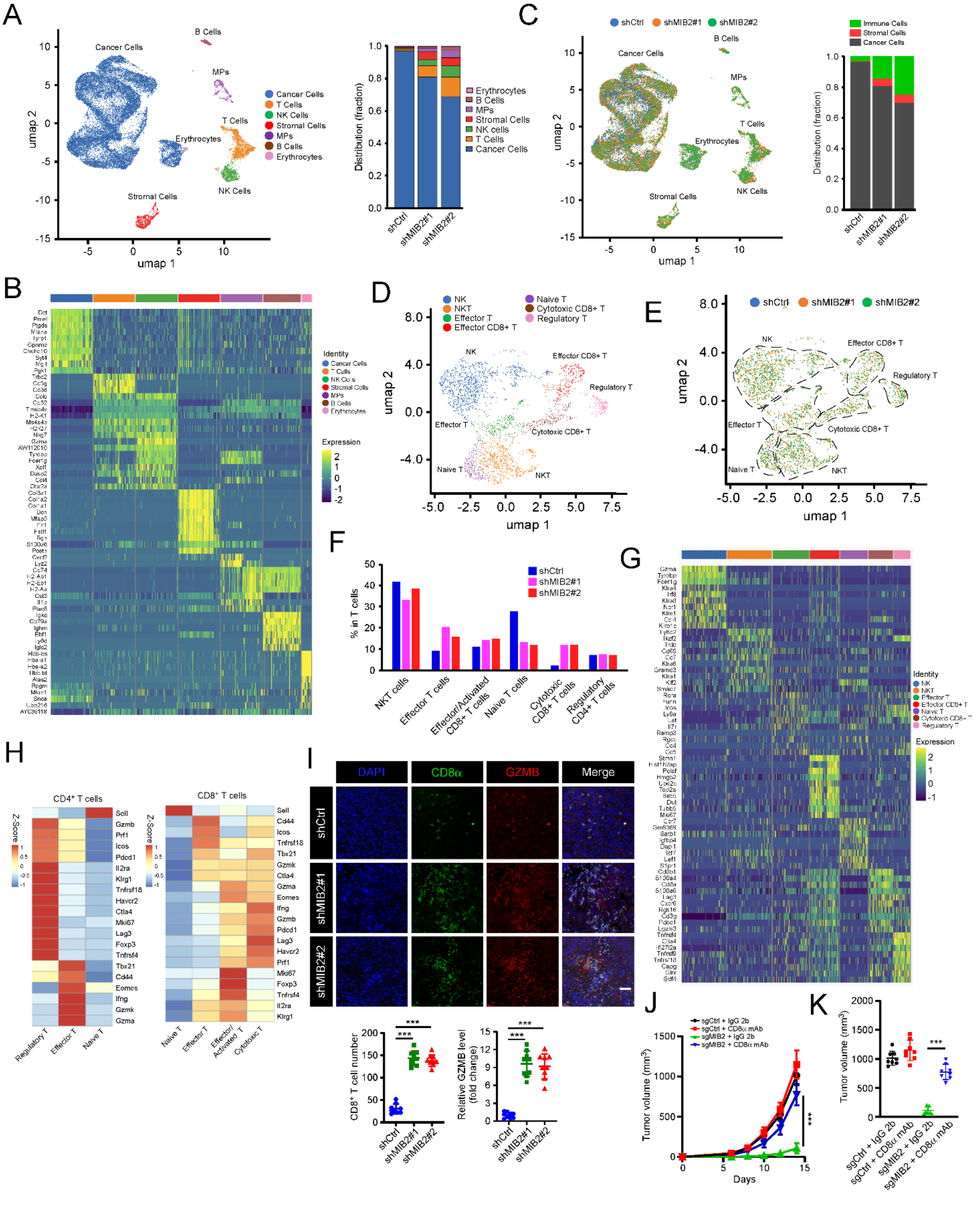

Figure S4. scRNA-seq analysis identifies tumor-infiltrating lymphocytes. A, Uniform Manifold Approximation and Projection (UMAP) plot showing cell populations in tumors from shCtrl and shMIB2 tumor cells (left). Distribution fraction of identified cancer, stromal, and immune cells in shCtrl and shMIB2 groups (right). **B**, Heatmap showing the expression of selected markers in T cell clusters. **C**, UMAP plot showing the cell distribution within identified cell populations in shCtrl and shMIB2 groups (left). Distribution fraction of identified cancer, stromal, and immune cells in

shCtrl and shMIB2 groups (right). **D**, UMAP plot displaying the subpopulations of T cells identified in **A**. **E**, UMAP plot showing the T cell distribution within identified cell populations in shCtrl and shMIB2 groups. **F**, Percentage of different tumor-infiltrating T cell populations identified in **D**. **G**, Heatmap showing the expression of selected markers for all cell clusters. **H**, Heatmap showing the expression of selected markers in $CD4^+$/$CD8^+$ T cell clusters. **I**, Immunostaining of CD8 and granzyme B (GZMB) in tumors. Data are represented as mean ± SD (n = 9); 3 tissue slides per tumor. Left: representative images; Scale bar, 50 µm. Right: quantification. **J,K**, Growth curves (**J**) and tumor volumes (**K**) of B16-F10 syngeneic tumors treated with control antibody or CD8α mAb (n = 8 per group). ***, p<0.001, by 1-way ANOVA among 3 groups. Data indicate the mean ± SD.

Supplemental Figure 5

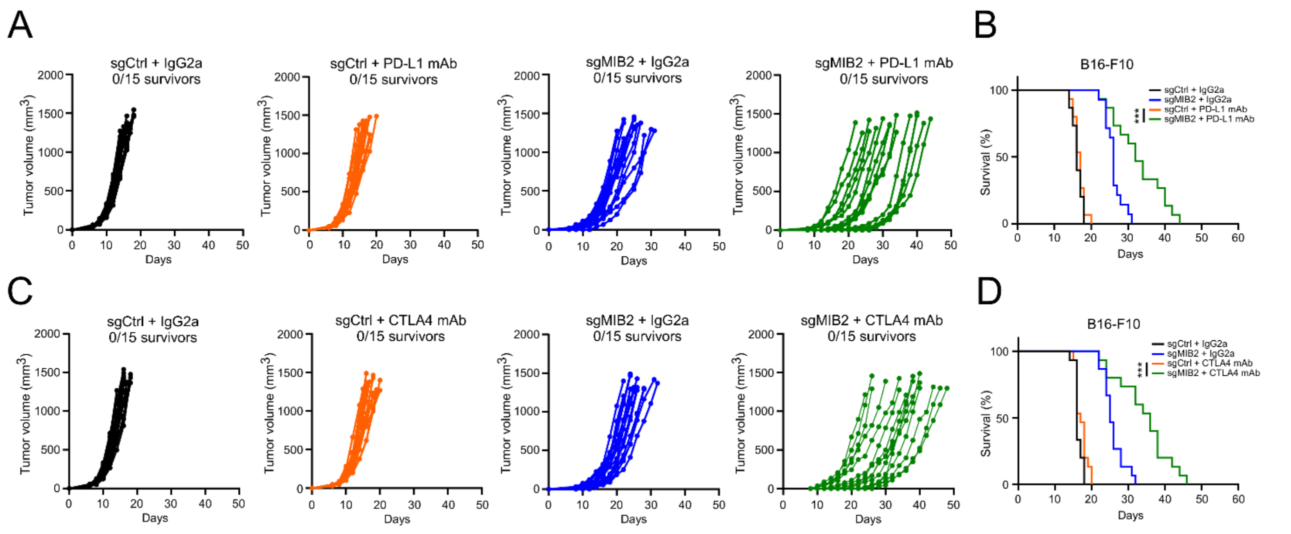

Figure S5. Depletion of MIB2 improves the efficacy of anti-PD-L1 and anti-CTLA4 immunotherapies. **A**, Volumes of B16-F10 syngeneic tumors treated with control antibody (IgG2a) or PD-L1 mAb. **B**, Kaplan–Meier survival curves for each treated group from **A** (n = 15 per group). ***, p<0.001. **C**, Volumes of B16-F10 syngeneic tumors treated with control antibody or CTLA4 mAb. **D**, Kaplan–Meier survival curves for each treated group from **C** (n = 15 per group). ***, p<0.001, by 1-way ANOVA among 3 groups. Data indicate the mean ± SD.

Supplemental Figure 6

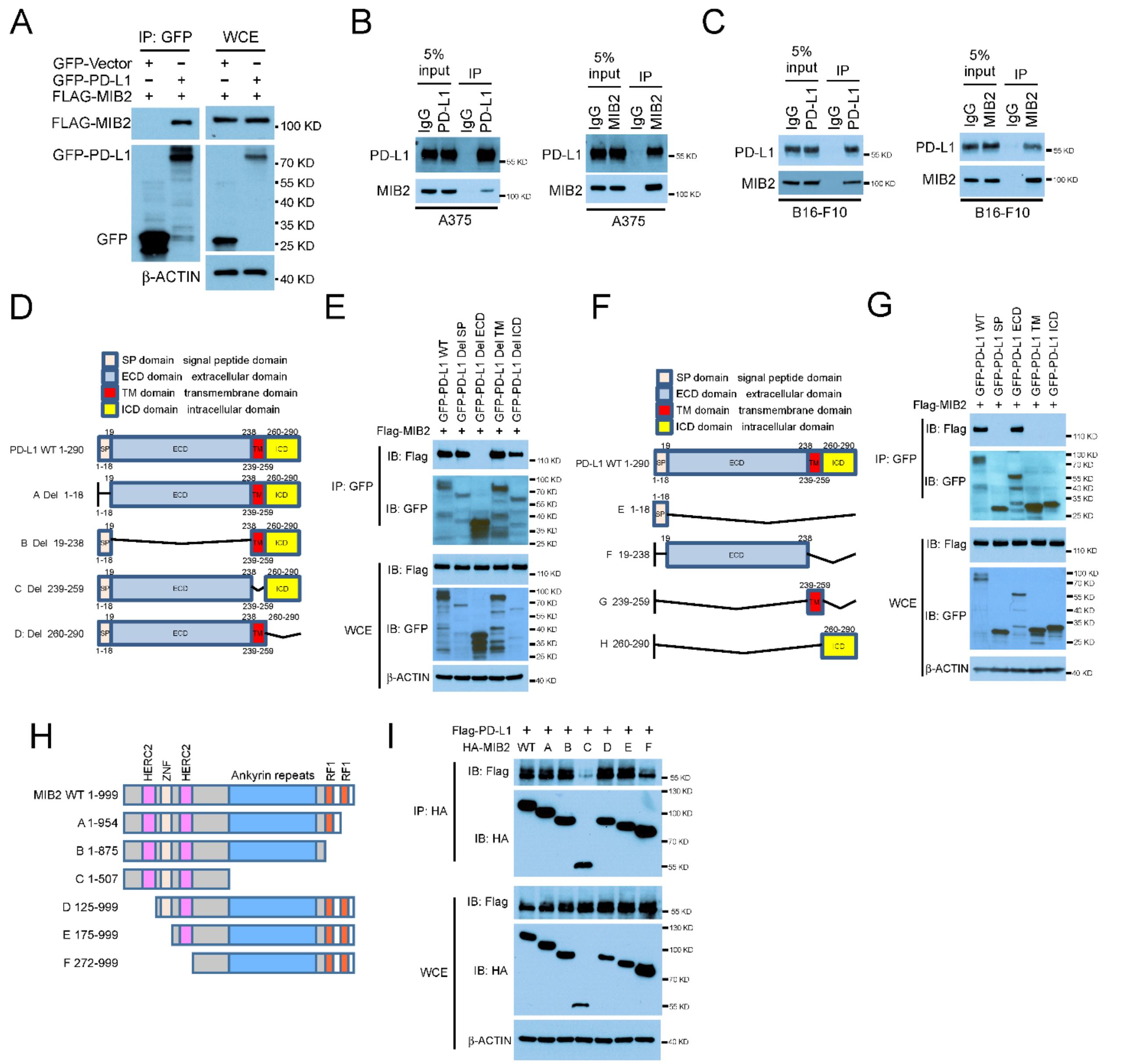

Figure S6. MIB2 interacts with PD-L1. A, Co-immunoprecipitation (Co-IP) analysis of PD-L1 and MIB2 in 293T cells transfected with the indicated constructs. **B,C**, Co-IP analysis of the endogenous PD-L1 and MIB2 interaction in A375 (**B**) and B16-F10 (**C**) cells. **D-G,** Deletion mutants of PD-L1. FLAG-MIB2 was co-transfected with GFP-PD-L1 mutant constructs into 293T cells for 48 h, the interaction between MIB2 and truncated PD-L1 was examined by Co-IP analysis. SP, signal peptide domain; ECD, extracellular domain; TM, transmembrane domain; ICD, intracellular domain. **H,I**, Deletion mutants of MIB2. FLAG-PD-L1 was co-transfected with HA-MIB2 mutant constructs into 293T cells for 48 h, the interaction between PD-L1 and truncated MIB2 was examined by Co-IP analysis. HERC2, the HECT and RLD Domain Containing E3 Ubiquitin Protein Ligase 2 (HERC2) domain; ZNF, zinc finger domain; Ankyrin repeats, ankyrin repeats domain; RF1 and RF2, ring finger domains 1 and 2.

Supplemental Figure 7

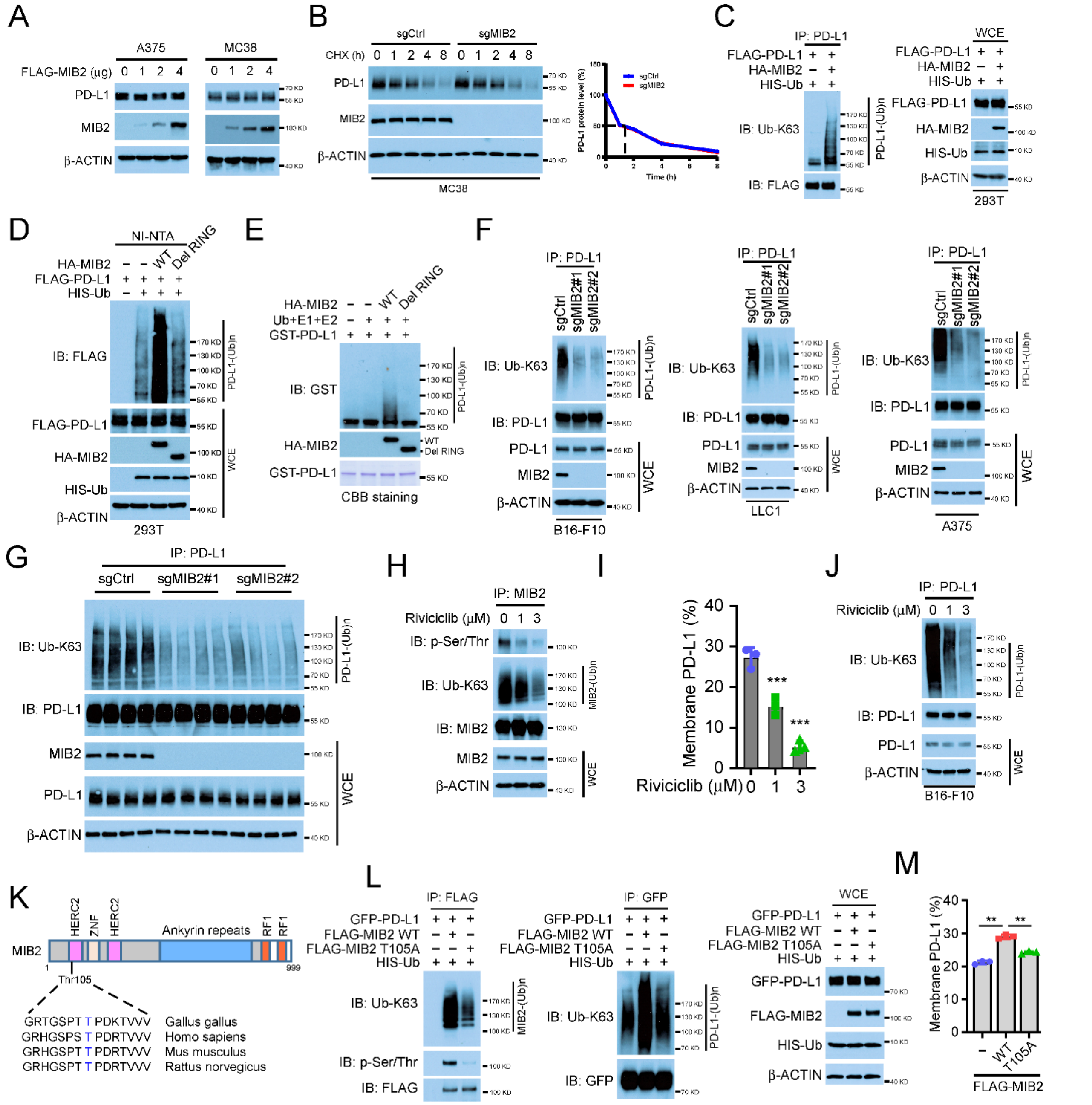

Figure S7. MIB2 phosphorylation promotes the E3 ligase activity. A, IB analysis of A375 and MC38 cells with exogenous FLAG-MIB2. **B**, IB analysis of PD-L1 protein levels in MC38 cells upon MIB2 KO and treatment with 30 ug/mL cycloheximide (CHX). **C**, IP and IB analysis of 293T cells transfected with expression constructs as indicated. WCE was subjected to PD-L1 ubiquitination analysis with Ub-K63 antibody. **D**, IP and IB analysis of PD-L1 ubiquitination in 293T cells transfected with HA-MIB2 WT or RING domain deletion mutant (HA-MIB2 Del RING). **E**, In vitro PD-L1 ubiquitination by MIB2 in a cell-free system. **F**, Ubiquitination analysis of endogenous PD-L1 in B16-F10, LLC1, and A375 cells with sgCtrl or sgMIB2 using Ub-K63 antibody. **G**, PD-L1 ubiquitination analysis of B16-F10 tumors from C57BL/6 mice. **H,** IP and IB analysis of MIB2 phosphorylation/ubiquitination with Ub-K63 antibody in B16-F10 cells treated with Riviciclib. **I**, FACS analysis of PD-L1 in B16-F10 cells treated with CDK1 inhibitor Riviciclib. **J**, IP and IB analysis of PD-L1 ubiqtuination with Ub-K63 antibody in B16-F10 cells treated with Riviciclib. **K**, Alignment of conserved MIB2 Thr105 residues among different species. **L**, IP and IB analysis of 293T cells transfected with expression constructs as indicated. WCE was subjected to MIB2 phosphorylation and MIB2/PD-L1 ubiquitination analysis with Ub-K63 antibody. **M**, FACS analysis of PD-L1 in B16-F10 cells expressing MIB2 WT or T105A. **, $p<0.01$; ***, $p<0.001$, by 1-way ANOVA among 3 groups. Data indicate the mean ± SD.

Supplemental Figure 8

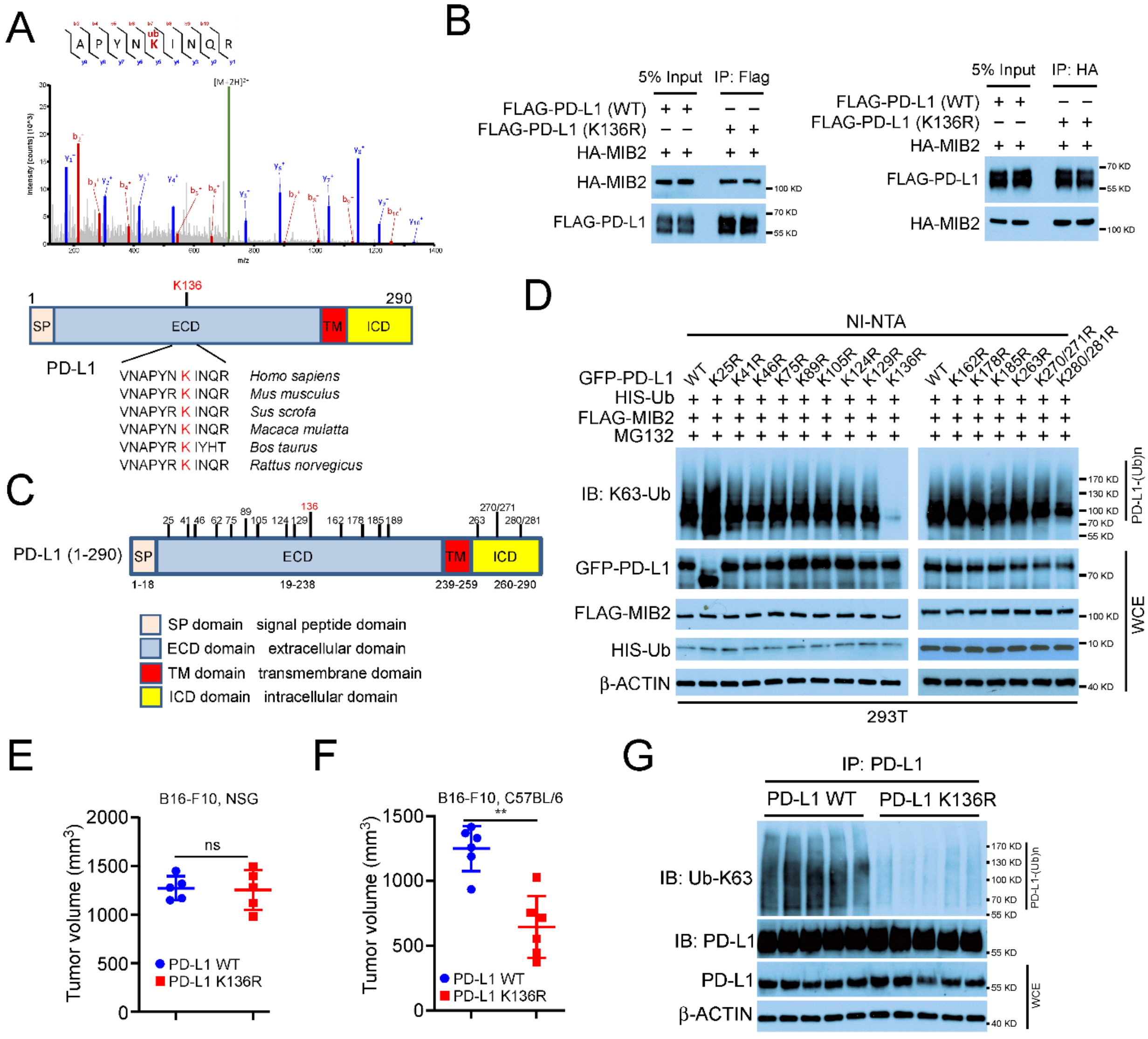

Figure S8. MIB2 promotes PD-L1 K136 ubiquitination. A, MS identification of the PD-L1 ubiquitination site by MIB2 (Top). Alignment of conserved PD-L1 K136 residue among different species (Bottom). **B**, Co-IP analysis of PD-L1 (WT or K136R) and MIB2 interaction in 293T cells transfected with the indicated constructs. **C**, A schematic of PD-L1 showing all potential lysine residues. **D**, *In vivo* PD-L1 ubiquitination by MIB2 in 293T cells transfected with various PD-L1 mutants. **E**,**F**, Tumor volumes of NSG (**E**) or C57BL/6 (**F**) mice inoculated with B16-F10 cells expressing PD-L1 WT or the K136R mutant (n = 5 per group). ns, not statistically significant, **, p<0.01, by unpaired, 2-tailed t test between 2 groups (**E** and **F**). Data indicate the mean ± SEM. **G**, PD-L1 ubiquitination analysis of B16-F10 tumors expressing PD-L1 WT or the K136R from C57BL/6 mice.

Supplemental Figure 9

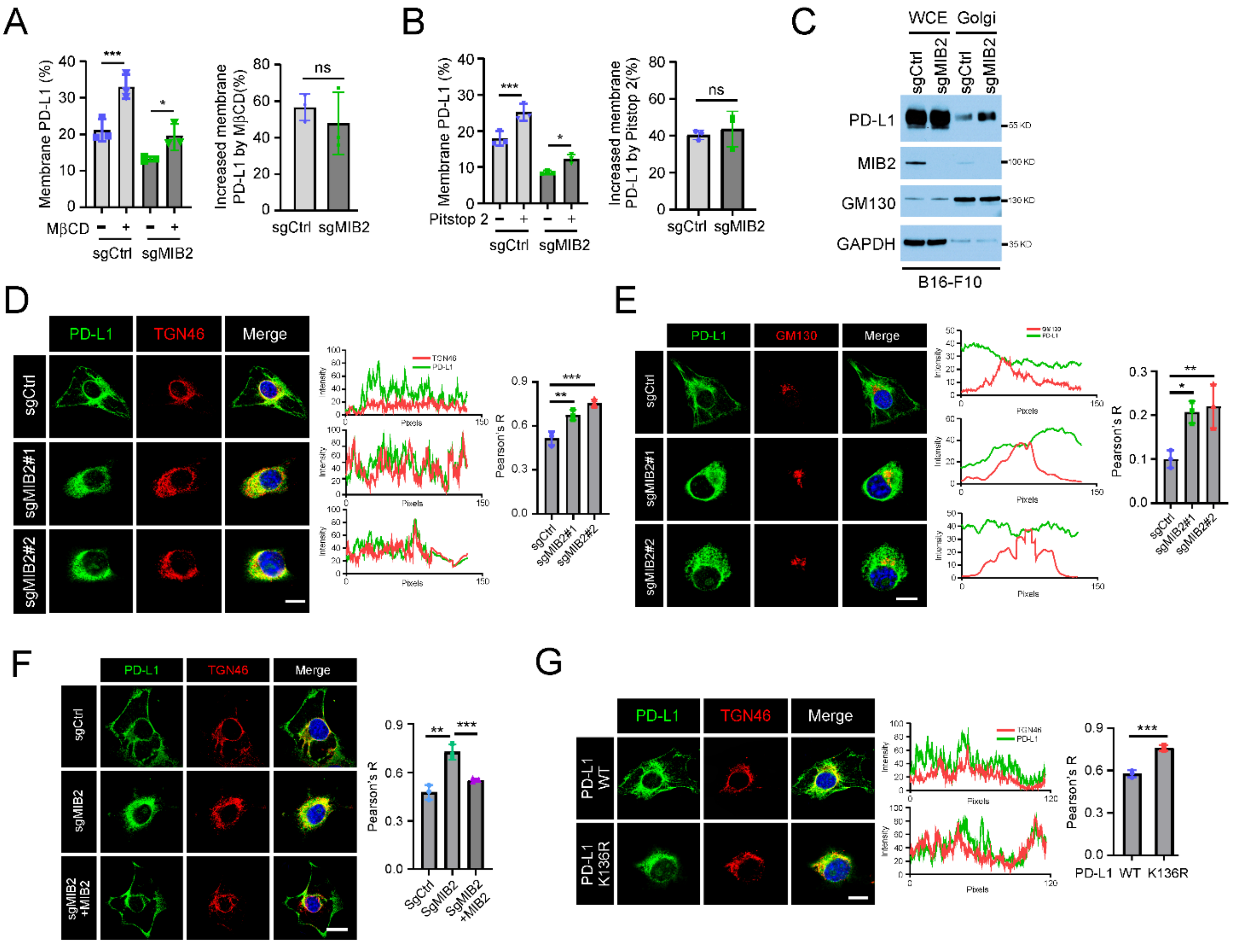

Figure S9. MIB2 is required for PD-L1 exocytosis. A,B,FACS analysis of PD-L1 expression in B16-F10 (sgCtrl and sgMIB2) stable cells treated with MβCD (**A**) or Pitstop 2 (**B**). **C**, IB analysis of PD-L1 in the WCE and isolated Golgi from B16-F10 cells. **D,** Co-localization of PD-L1 and Golgi markers TGN46 in MC38 cells. Left: representative images; Scale bar, 10 µm. Middle, the intensity profiles of PD-L1 and TGN46; Right, Pearson's R-value graph; (n = 3 independent experiments). **E**, Co-localization of PD-L1 and Golgi markers GM130 in MC38 cells. Left: representative images; Scale bar, 10 µm. Middle, the intensity profiles of PD-L1 and GM130; Right, Pearson's R-value graph; means ± SD (n = 3 independent experiments). **F**, Immunostaining of PD-L1 and TGN46 in MC38 cells. Left: representative images; Scale bar, 10 µm. Right, Pearson's R-value graph; means ± SD (n = 3 independent experiments). G**,** Co-localization of PD-L1 (WT or K136R) and TGN46 in MC38 cells. Left: representative images; Scale bar, 10 µm; Middle, the intensity profiles of PD-L1 and TGN46; Right, Pearson's R-value graph; (n = 3 independent experiments). *, p<0.05; **, p<0.01; ***, p<0.001, by unpaired, 2-tailed t test between 2 groups; and by 1-way ANOVA among 3 groups. ns, not statistically significant. Data indicate the mean ± SD.

Supplemental Figure 10

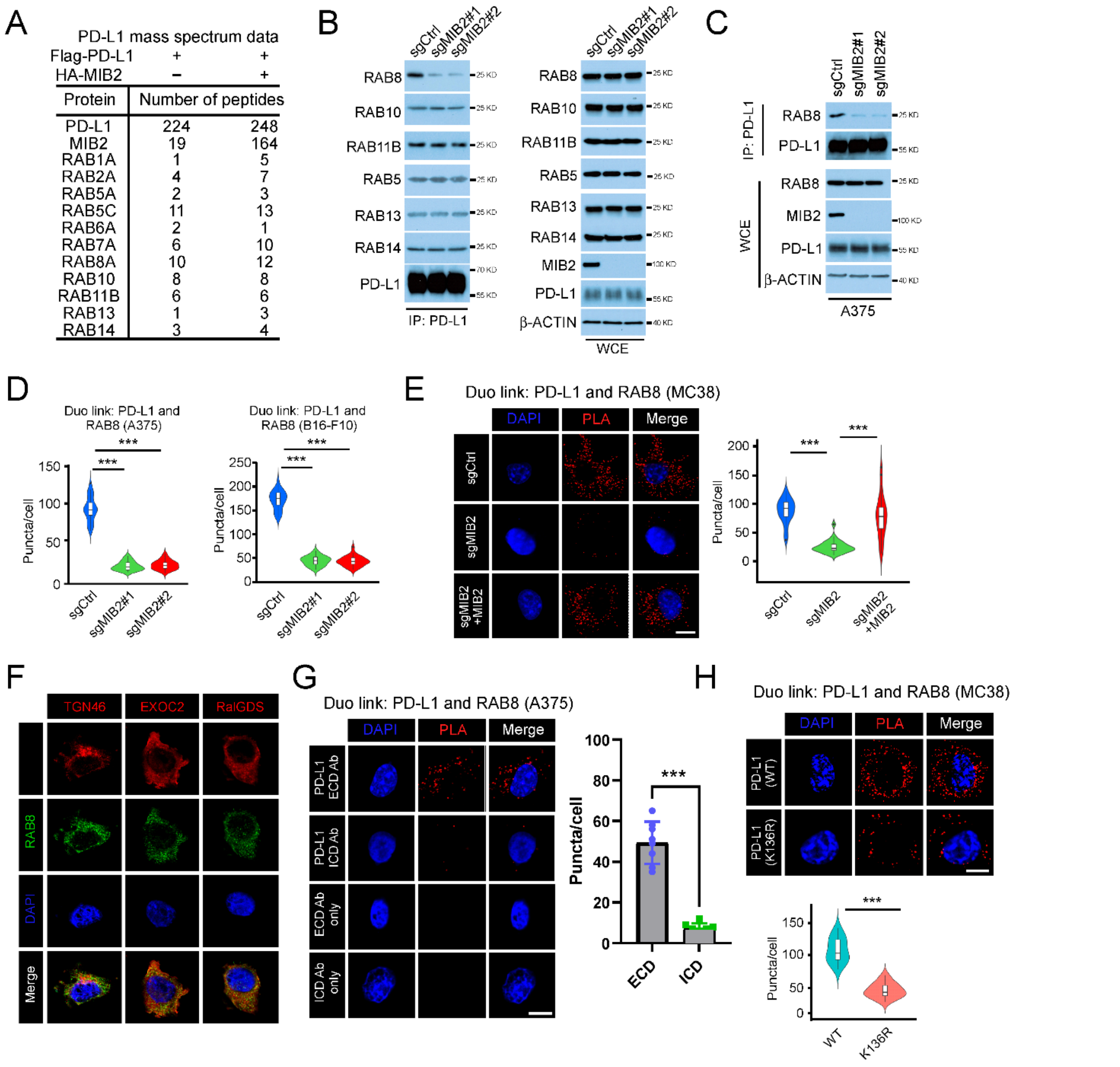

Figure S10. PD-L1 binds RAB8. A, List of RAB family proteins identified by MS analysis. **B**, IP and IB analysis of endogenous PD-L1 and its interacting RAB proteins in MC38 cells. **C**, IP and IB analysis of endogenous PD-L1 and its interacting RAB8 in A375 cells. **D**, PLA analysis of PD-L1 and RAB8 interaction in A375 and B16-F10 cells. **E**, PLA analysis of PD-L1 and RAB8 interaction in MC38 cells. Left: representative images. Scale bar, 10 μm. Right: quantitation. **F**, Immunostaining of RAB8 and TGN46, EXOC2, or RalGDS in B16-F10 cells. **G**, PLA analysis of PD-L1 and RAB8 interaction in A375 cells with antibodies specific for ECD and ICD of PD-L1. Left: representative images. Scale bar, 10 μm. Right: quantitation. **H**, PLA analysis of PD-L1 (WT or K136R) and RAB8 interaction in MC38. Top: representative images; Scale bar, 10 μm. Bottom: quantitation. Lines within the boxes denote median values, the box top is the upper quartile (75th percentile) and box bottom is the lower quartile (25th percentile), widths denote cell densities. ***, $p<0.001$, by unpaired, 2-tailed t test between 2 groups; and by 1-way ANOVA among 3 groups. ns, not statistically significant. Data indicate the mean ± SD.

Supplemental Figure 11

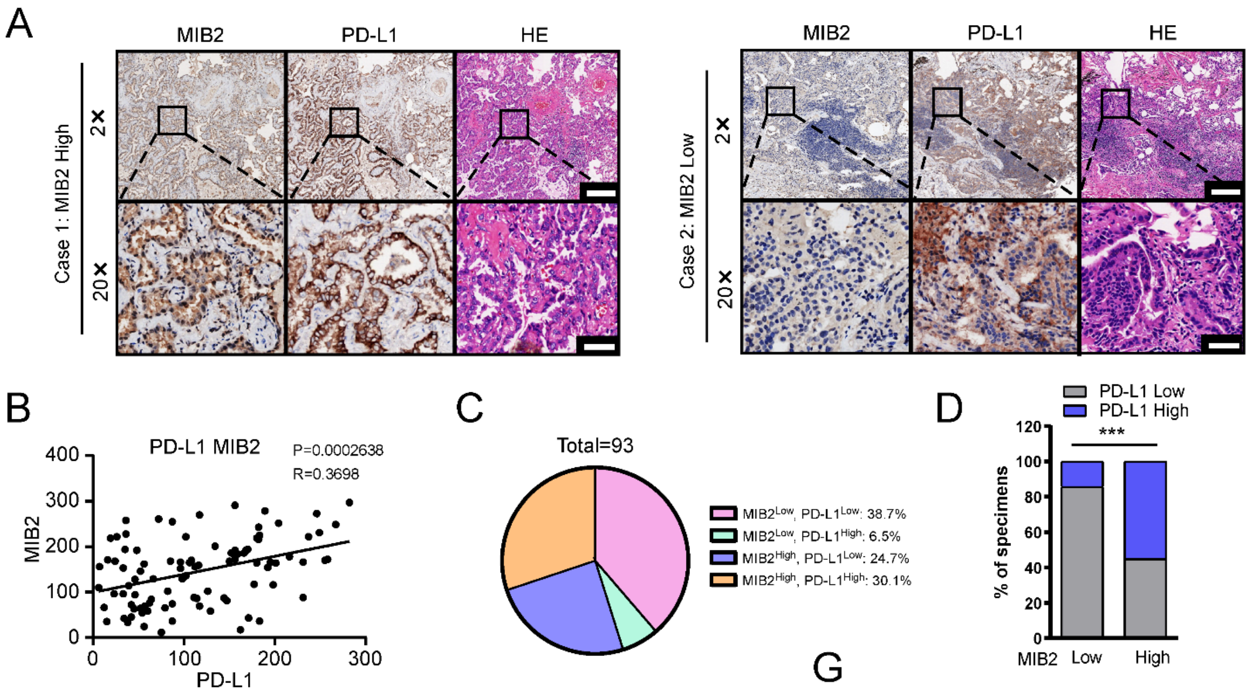

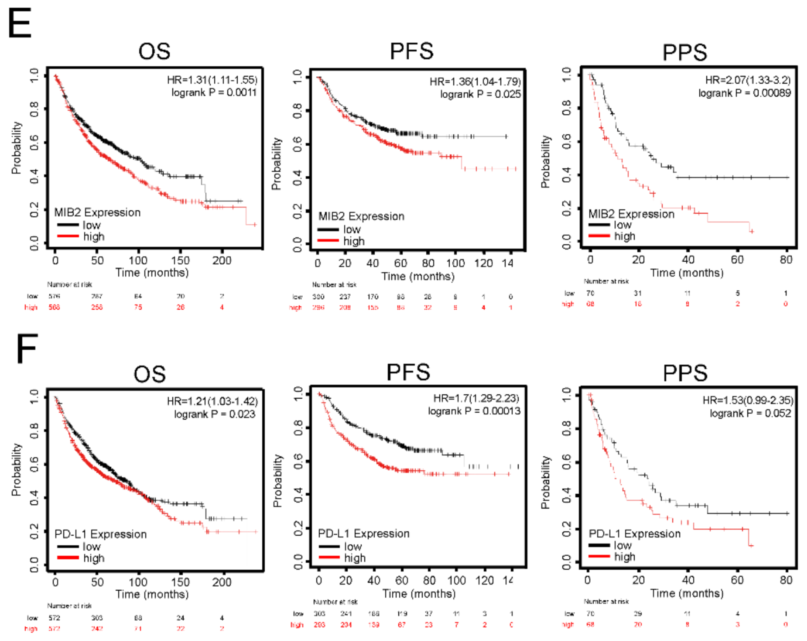

G

PD-L1&MIB2 Predicted

		PD	PR	SD	Total
Actual	PD	9	2	2	13
	PR	3	6	2	11
	SD	4	1	2	7
	Total	16	9	6	31
	Accuracy	56.2%	66.7%	33.3%	54.8%

MIB2 Predicted

		PD	PR	SD	Total
Actual	PD	10	3	0	13
	PR	6	5	0	11
	SD	6	1	0	7
	Total	22	9	0	31
	Accuracy	45.5%	55.6%	NA	38.8%

PD-L1 Predicted

		PD	PR	SD	Total
Actual	PD	8	3	2	13
	PR	10	1	0	11
	SD	7	0	0	7
	Total	25	4	2	31
	Accuracy	32.0%	25.0%	0.0%	22.3%

Figure S11. PD-L1 membrane protein level positively correlates with MIB2 expression in NSCLC. **A**, Representative images of IHC staining for MIB2 and PD-L1 from our NSCLC cohort. Left, a case with high-level MIB2; Right, a case with low-level MIB2. Scale bar, top: 200 µm; bottom: 50 µm. **B**, Scatterplot showing the correlation between MIB2 and PD-L1 membrane protein levels in our NSCLC specimens. **C**, The pie chart of the expression of MIB2 and PD-L1 membrane protein levels in our 93 NSCLC specimens. **D**, The percentage of specimens displaying low or high PD-L1 membrane levels compared to MIB2 in our cohort. **E,F**, Kaplan–Meier curves of the relationship between survival and MIB2 (**E**) or PD-L1 (**F**) mRNA levels in published NSCLC cohorts. OS, Overall Survival; PFS, Progression-free survival; PPS, Post-progression Survival. **G**, A table summarizing the accuracies of MIB2+PD-L1, MIB2 or PD-L1 single predicted treatment responses from the Bayes Model. ***, $p<0.001$ by $\chi 2$ test for contingency in D.

Table S1: E3 ligase screened by shRNA knockdown

Number	Gene
1	*BTRC*
2	*DTX1*
3	*ARIH1*
4	*CUL5*
5	*HERC2*
6	*STUB1*
7	*TRIP12*
8	*CUL4B*
9	*RING1*
10	*RNF14*
11	*TRIM27*
12	*UBE3C*
13	*TRCP*
14	*DTX2*
15	*RLIM*
16	*TRAF2*
17	*PPIL2*
18	*ANAPC2*
19	*SMURF2*
20	*CUL2*
21	*FBXL21*
22	*RBCK1*
23	*FBXO7*
24	*HACE1*
25	*UHIF1*
26	*HERPUD1*
27	*RNF186*
28	*UBOX5*
29	*ZBTB16*
30	*ANAPC10*
31	*FBXO24*
32	*UBR1*
33	*SKP1*
34	*ANAPC4*
35	*KIAA0317*
36	*CAND1*
37	*PARK2*
38	*ANAPC5*
39	*UBR2*
40	*MIB1*
41	*TRIM34*
42	*FBXW8*
43	*ITCH*
44	*NAE1*
45	*TRIM22*
46	*FBXW11*
47	*RNF40*
48	*RNF5*
49	*FBXW5*
50	*PJA1*
51	*G2E3*
52	*RFWD2*
53	*RNF144A*
54	*FBXO3*
55	*HERC3*
56	*SMURF1*
57	*RNF138*
58	*HERC5*
59	*FBXO27*
60	*HECW2*
61	*AMFR*
62	*UHIF2*
63	*RNF19A*
64	*SKP2*
65	*RNF125*
66	*FBXO17*
67	*TRIM32*
68	*CDC23*
69	*RNF112*
70	*HERC1*
71	*RNF148*
72	*USP7*
73	*ZNRF2*
74	*FBXO6*
75	*LNX2*
76	*TRIM5*
77	*FBXO25*
78	*FBXO15*
79	*RNF128*
80	*TCEB1*
81	*RNF130*
82	*RNF152*
83	*FBXO5*
84	*FBXO11*
85	*TRIM50*
86	*HECTD2*
87	*SENP7*
88	*TRIM40*
89	*BIRC6*
90	*RNF132*
91	*CBX8*
92	*FBXL6*
93	*CDC34*
94	*CUL7*
95	*C12ORF51*
96	*RNF175*
97	*PHF21B*
98	*RNF216*
99	*RNF25*
100	*LMO7*
101	*WWP1*
102	*TRAF5*
103	*TRIM26*
104	*CUL1*
105	*FBXL2*
106	*UBE3B*
107	*TRIM29*
108	*PBPF19*
109	*CCNF*
110	*FBXO2*
111	*WWP2*
112	*RNF2*
113	*TRAF6*
114	*NEDD8*
115	*RNF8*
116	*FBXL3*
117	*PML*
118	*SIAH1*
119	*BMI1*
120	*FBXW2*
121	*RNF121*
122	*UBE3A*
123	*BIRC2*
124	*TRIM25*
125	*FBXL5*
126	*FBXO2*
127	*CUL4A*
128	*CHFR*
129	*SIAH2*
130	*CNDT4*
131	*UBE4A*
132	*BIRC3*
133	*TRIM3*
134	*FBXW7*
135	*MIB2*
136	*BARD1*
137	*RBX1*

Table S2: Protein expression of MIB2 and PD-L1 in 93 cases of NSCLC tissues.

		PD-L1			*p* value
		**Low**	**High**	**Total**	
	**Low**	36	6	42	
**MIB2**	**High**	23	28	51	<0.001
	**Total**	59	34	93	

χ^2 test. $p<0.05$ indicates a significant association among the variables.

Table S 3 NSCLC patients with needle biopsy

Patient No.	Gender	Age	Tumor types	TNM stage	Response
1	Male	69	LUSC	T4N2M1c	PR
2	Male	79	LUSC	T3N2M0	PR
3	Male	58	LUSC	T2aN0M0	PD
4	Male	66	LUSC	T4N3M1c	SD
5	Male	65	LUSC	T4N3M1c	PD
6	Male	56	LUSC	T1N3M0	PR
7	Male	68	LUSC	T3N2M1b	PR
8	Male	56	LUSC	T2aN1M0	PD
9	Male	68	LUSC	T4N3M1a	PD
10	Female	67	LUSC	T2N3M1c	PD
11	Male	70	LUSC	T4N2M1c	PD
12	Male	53	LUSC	T4N3M0	SD
13	Male	68	LUSC	T2N3M0	PD
14	Male	74	LUSC	T3N1M0	PR
15	Male	65	LUSC	T2bN2M1a	PD
16	Male	61	LUSC	T2N0M0	SD
17	Male	67	LUSC	T3N0M0	PR
18	Female	56	LUSC	T4N2M1c	PD
19	Male	62	LUSC	T2N3M0	PD
20	Male	57	LUSC	T4N2M1b	PR
21	Male	60	LUSC	T4N3M1c	PR
22	Male	66	LUSC	T4N3M1a	PD
23	Male	60	LUSC	T4N3M1a	SD
24	Male	73	LUAD	T4N3M1c	PD
25	Female	39	LUAD	T2aN3M1C	PD
26	Male	84	LUAD	T3N0M0	SD
27	Male	63	LUAD	T2N0M0	SD
28	Male	84	LUAD	T2aN3M1C	SD
29	Female	64	LUAD	T4N0M1A	PR
30	Male	66	LUAD	T2aN2M1a	PR
31	Male	61	LUAD	T4N3M1a	PR

LUAD, lung adenocarcinoma; LUSC: lung squamous cell carcinoma. Patients had partial response (PR), stable disease (SD), or progressive disease (PD). Patients were stratified into response groups based on RECIST 1.1 criteria. Patients with PR > 3 months were classified as responders, while patients with SD ≤ 3 months or PD were classified as non-responders.
